# Modification of tRNA^Lys^_UUU_ by Elongator Is Essential for Efficient Translation of Stress mRNAs

**DOI:** 10.1371/journal.pgen.1003647

**Published:** 2013-07-18

**Authors:** Jorge Fernández-Vázquez, Itzel Vargas-Pérez, Miriam Sansó, Karin Buhne, Mercè Carmona, Esther Paulo, Damien Hermand, Miguel Rodríguez-Gabriel, José Ayté, Sebastian Leidel, Elena Hidalgo

**Affiliations:** 1 Oxidative Stress and Cell Cycle Group, Departament de Ciències Experimentals i de la Salut, Universitat Pompeu Fabra, Barcelona, Spain; 2 Max Planck Research Group for RNA Biology, Max Planck Institute for Molecular Biomedicine, Münster, Germany; 3 Namur Research College (NARC), The University of Namur, Namur, Belgium; 4 Centro de Biología Molecular “Severo Ochoa”, Universidad Autónoma de Madrid (UAM), Consejo Superior de Investigaciones Científicas (CSIC), Madrid, Spain; University of California San Francisco, United States of America

## Abstract

The Elongator complex, including the histone acetyl transferase Sin3/Elp3, was isolated as an RNA polymerase II-interacting complex, and cells deficient in Elongator subunits display transcriptional defects. However, it has also been shown that Elongator mediates the modification of some tRNAs, modulating translation efficiency. We show here that the fission yeast Sin3/Elp3 is important for oxidative stress survival. The stress transcriptional program, governed by the Sty1-Atf1-Pcr1 pathway, is affected in mutant cells, but not severely. On the contrary, cells lacking Sin3/Elp3 cannot modify the uridine wobble nucleoside of certain tRNAs, and other tRNA modifying activities such as Ctu1-Ctu2 are also essential for normal tolerance to H_2_O_2_. In particular, a plasmid over-expressing the tRNA^Lys^_UUU_ complements the stress-related phenotypes of Sin3/Elp3 mutant cells. We have determined that the main H_2_O_2_-dependent genes, including those coding for the transcription factors Atf1 and Pcr1, are highly expressed mRNAs containing a biased number of lysine-coding codons AAA versus AAG. Thus, their mRNAs are poorly translated after stress in cells lacking Sin3/Elp3 or Ctu2, whereas a mutated *atf1* transcript with AAA-to-AAG lysine codons is efficiently translated in all strain backgrounds. Our study demonstrates that the lack of a functional Elongator complex results in stress phenotypes due to its contribution to tRNA modification and subsequent translation inefficiency of certain stress-induced, highly expressed mRNAs. These results suggest that the transcriptional defects of these strain backgrounds may be a secondary consequence of the deficient expression of a transcription factor, Atf1-Pcr1, and other components of the transcriptional machinery.

## Author Summary

The success of a biological event such as cellular adaptation to environmental changes requires the complex process of protein expression to be carried out with high efficiency and fidelity. Thus, not only transcription but also mRNA homeostasis and translation have to be performed with maximum efficiency, or survival would be hampered. Our study demonstrates that the role of Elongator, a putative Pol II-associated complex, in survival to stress is to optimize translation efficiency by modifying some particular tRNAs. We show here that Sin3/Elp3, an Elongator component, participates in the modification of the anticodon of the low copy number tRNA^Lys^_UUU_, which probably favours codon recognition. This tRNA recognizes one of the two codons for lysine, which is down-represented in highly expressed constitutive genes. The stress mRNAs, highly-expressed upon stress conditions, have not adapted their lysine codon usage from AAA-to-AAG, and proper tRNA^Lys^_UUU_ modification by Elongator is an alternative strategy to accomplish efficient translation of these AAA-containing, abundant stress mRNAs.

## Introduction

Unicellular organisms are particularly exposed to the environment, and the major changes in microbial gene expression programs arise as a consequence of extracellular stresses. Most regulation is achieved by transcriptional events, with a shift of the transcriptional machinery from growth- to stress-related genes. Therefore, the classical large complexes which contribute to a strong and efficient RNA polymerase II (Pol II) gene transcription, such as Mediator and SAGA, do contribute to stress survival, and genetic defects in non-essential components of these complexes can render phenotypes of sensitivity to stress.

In fission yeast, the MAP kinase Sty1 pathway is essential to induce massive changes in the gene expression programs in response to environment insults (for reviews, see [1], [2]). Upon different types of life-threatening insults such as osmotic or oxidative stress, heat shock or nutrient deprivation, a cascade of phosphorylations results in the activation of Sty1, which then accumulates in the nucleus and triggers a broad transcriptional change of up to 5–10% of the genome. Thus, hundreds of genes become repressed, while hundreds of others are activated, to promote survival. These genes, positively or negatively controlled by different stresses in a Sty1-dependent manner, were called CESR (core environmental stress response) genes [3]. The main effector of such transcriptional events, or at least the activation ones, is the heterodimeric transcription factor Atf1-Pcr1 [3], [4]. Several activities modulating chromatin accessibility and compactness modulate the Sty1-dependent transcription program (for a review, see [5]). For instance, the absence of the histone acetyl transferase (HAT) and SAGA component Gcn5 renders cells sensitive to several stresses due to defective chromatin remodelling along the stress genes [6], [7].

Thus, regarding the fission yeast stress response it is plausible to hypothesize that strains defective in chromatin remodeling activities and/or in components of the large complexes which contribute to an efficient Pol II transcription may be sensitive to stress. That would be the case of the transcription complex named Elongator, isolated in *Saccharomyces cerevisiae* as essential to trigger chromatin remodeling [8]. This multi-component complex includes Elp3, a HAT that regulates the levels of histone H3 lysine (Lys) 14 and H4 Lys8 acetylation [9]. A number of reports described the participation of Elongator in chromatin modulation, and strains devoid of some of its six components exhibit a pleiotropic phenotype, including transcriptional elongation defects and problems with polarized exocytosis (for a review, see [10]).

In 1985, an *S. pombe* strain named *sin3-193* was reported to have defects in transfer RNA (tRNA) modification, since digestion of tRNAs from this mutant strain to nucleosides and subsequent nucleoside analysis demonstrated the absence of one particular modification in uridine (U) [11]. The laboratory of Bystrom identified in 2005 the *sin3/elp3* gene product as one of the Elongator components, and isolated an equivalent tRNA modifying regulatory activity in *S. cerevisiae*'s Elp3 [12]. Later the same was found for Elongator from *Arabidopsis thaliana* [13] and *Caenorhabditis elegans* [14]. Importantly enough, most defects initially associated with a role of Elongator in transcription and exocytosis were bypassed by elevated levels of specific tRNAs, those normally modified by the complex (see below) [15], [16]. Thus, the diverse roles of Elongator are a matter of debate (for a review, see [17]).

The genetic code is degenerated, so that most amino acids are encoded by more than one triplet, some of which are more common than others and define the codon usage of a given organism. Up to 75–100 different post-transcriptional nucleoside modifications have been reported in eukaryotic tRNAs [18], many of which occur at the anticodon loop. In particular, a dual modification of a U (U_34_) at the 5′ wobble position of the anticodon of several tRNAs [those coding for glutamine (Gln), Lys and glutamic acid (Glu), having a UUB (‘B’ being G, C or U) anticodon] has been suggested to have a role in either translation fidelity [19]–[21] or efficiency [22]–[25], and to even be required for viability in yeast [26]. In *S. cerevisiae*, these modifications consist in the addition of a methoxycarbonylmethyl at carbon 5 of U by the Elongator complex (mcm^5^U_34_), and in a thiolation at carbon 2 by the Nfs1-Uba4-Urm1-Ncs6-Ncs2 network (s^2^U_34_) [15], [26]–[32]. As indicated above, Sin3/Elp3 of *S. pombe* has been reported to be required to generate the mcm^5^s^2^U modification in tRNAs [11], [12], and Ctu1-Ctu2 are the sequence and functional homologs of Ncs6-Ncs2 [33]. An *S. pombe* strain lacking both tRNA-modifying activities has recently been shown to display cell cycle defects [34].

In a genetic search for deletion mutants with altered sensitivity to H_2_O_2_, we have isolated the putative histone H3 HAT Sin3/Elp3, a component of the Elongator complex. Our initial assumption was that mutations in a chromatin-modifying activity such as Elongator should result in cells displaying stress sensitivity due to defects in transcriptional efficiency. However, our results indicate that Sin3/Elp3 mutant does not display enough alterations in transcriptional events as to explain the substantial sensitivity to peroxides. In fact, the levels of acetylated H3 (total or associated to stress genes) are not significantly affected in *Δsin3/elp3* cells. Instead, the wobble U of tRNAs for Lys, Gln and Glu is not modified in cells lacking Sin3/Elp3. This defect in tRNA modification seems to be sufficient to cause the oxidative stress phenotype, since cells devoid of the second modification pathway, required for the formation of s^2^U_34_, such as Ctu1 or Ctu2, are also sensitive to stress. Furthermore, over expression of one of these tRNA species, tRNA^Lys^_UUU_, is sufficient to complement all the stress defects of cells lacking Sin3/Elp3. Importantly, we show here that the mRNAs for the Atf1 and Pcr1 transcription factors, which are critical for CESR gene expression and are enriched in the AAA codon for Lys, are not greatly affected in the knock-out strain (*Δsin3/elp3*). However, Atf1 and Pcr1 protein levels are severely decreased in *Δsin3/elp3* and *Δctu2* cells. Furthermore, a mutated *atf1* transcript with AAA-to-AAG lysine codons is efficiently translated in all strain backgrounds.

## Results

### Cells lacking Sin3/Elp3 are sensitive to H_2_O_2_, but only display minor transcriptional defects

**Figure 1:**
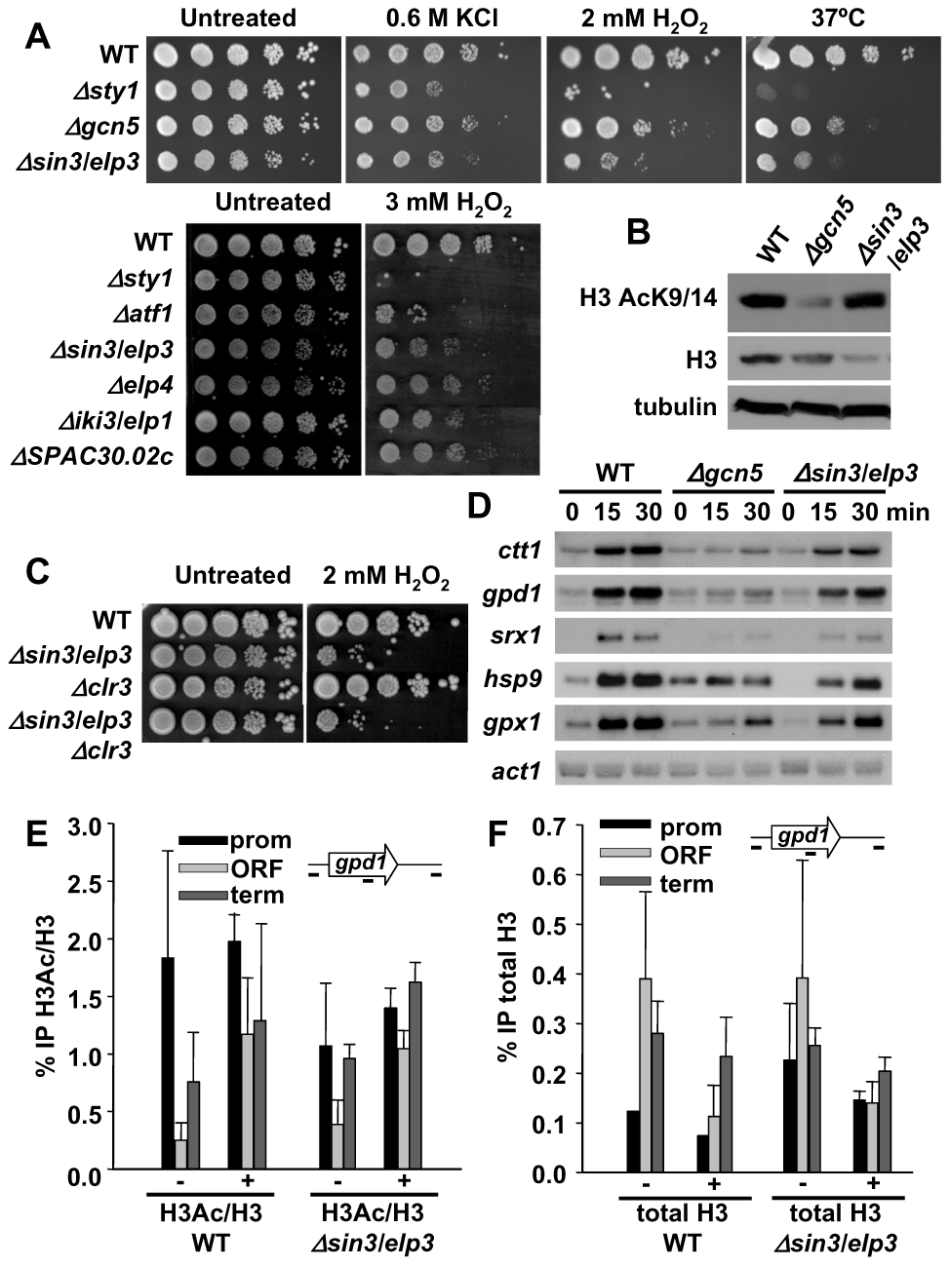
Cells lacking Sin3/Elp3 are sensitive to H_2_O_2_, but do not display major transcriptional defects. (A) *Δsin3/elp3* strain and other mutants of Elongator complex are sensitive to oxidative stress. Serial dilutions from cultures of strains 972 (WT), AV18 (*Δsty1*), MS161 (*Δgcn5*), IV16 (*Δsin3/elp3*), MS98 (*Δatf1*), IV72 (*Δelp4*), IV66 (*Δiki3/elp1*) and IV68 (*ΔSPAC30.02c; S. pombe* ortholog of *kti12*) were spotted onto rich plates without (Untreated) or with the indicated concentrations of H_2_O_2_ or KCl, and grown at 30°C unless indicated (37°C). (B) Total levels of histone H3 acetylation at lysines 9 and 14 are not affected in a *Δsin3/elp3* strain. Protein extracts from strains 972 (WT), MS161 (*Δgcn5*) and IV16 (*Δsin3/elp3*) were analyzed by Western blot with antibodies against acetylated Lys9 and Lys14 of histone H3 (H3K9K14-Ac) or total H3, as a loading control. (C) Deletion of *clr3* does not rescue *Δsin3/elp3* sensitivity to oxidative stress. Serial dilutions from cultures of strains 972 (WT), IV16 (*Δsin3/elp3*), SPK19 (*Δclr3*) and IV95 (*Δsin3/elp3 Δclr3*) were spotted onto rich plates without (Untreated) or with 2 mM H_2_O_2_. (D) Stress-dependent transcriptional analysis of wild-type and *Δsin3/elp3* cells. Culture of strains 972 (WT), MS161 (*Δgcn5*) and IV16 (*Δsin3/elp3*) were treated with 1 mM H_2_O_2_ for the indicated times. Total RNA was analyzed by Northern blot with probes for *ctt1, gpd1, hsp9, srx1*, and *gpx1*. *act1* is shown as a loading control. (E) Stress-dependent H3 acetylation at CESR genes does not require Sin3/Elp3. Cultures of strains 972 (WT) and IV16 (*Δsin3/elp3*) were treated (+) or not (−) with 1 mM H_2_O_2_ for 5 min. Chromatin immunoprecipitation (ChIP) assays were performed using antibodies specific for acetylated Lys9 and Lys14 of histone H3 (H3Ac) or against unmodified C-terminal domain of H3 (H3). The percentage of immunoprecipitation of acetylated H3 versus total H3 is indicated (% IP H3Ac/H3). ChIP experiments were performed using primers covering promoter (prom), coding (ORF) and termination (term) sequences of the *gpd1* gene. (F) Stress-dependent nucleosome eviction at CESR genes does not require Sin3/Elp3. The same experiment as in E is represented here as the percentage of immunoprecipitation of total H3 (%IP total H3). Error bars (SEM) for all ChIP experiments were calculated from biological triplicates.

Defects in activities required to mediate massive changes in gene expression should result in cells displaying stress sensitivity. On this basis, we screened a collection of *S. pombe* deletion strains searching for mutants with impaired survival against H_2_O_2_ on solid plates. We isolated several strains with defects in chromatin modifying activities, such as the HAT Gcn5 [7]. Another strain displaying even more severe growth sensitivity to peroxides is that lacking Sin3/Elp3, a HAT and component of the Elongator complex (Figure 1A). We analyzed mutants in other components of Elongator present in our deletion collection (Table S1), and at least subunits Elp4, Iki3/Elp1 and SPAC30.02c [the homolog to KTI12, an *S. cerevisiae* protein associated to Elongator [35]] are required for wild-type tolerance to H_2_O_2_ (Figure 1A).

Recently, it had been shown that Gcn5 is the major contributor to H3 acetylation in fission yeast, with only a very modest decrease in H3 acetylation at Lys9 and Lys14 in cells lacking Sin3/Elp3 [36] (Figure 1B). Furthermore, we could not suppress the *Δsin3/elp3* defects to H_2_O_2_ stress by further deletion of the stress-related histone H3 deacetylase Clr3 (Figure 1C). Finally, the expression levels and/or the induction kinetics of several H_2_O_2_–inducible genes are affected in *Δsin3/elp3* cells, but to a lesser extent than cells lacking the HAT Gcn5 (Figure 1D), even though the sensitivity to peroxides of *Δsin3/elp3* cells is more severe than that of *Δgcn5* cells (Figure 1A).

Next, we performed chromatin immunoprecipitation (ChIP) experiments in an attempt to detect Sin3/Elp3 at or close to stress genes, as previously found for Gcn5 [7], but we were unable to find Sin3/Elp3 associated with CESR genes (data not shown). Furthermore, we performed ChIP analysis of total and acetylated histone H3 to detect a localized effect of the lack of Sin3/Elp3 on the nucleosomes of the stress genes. However, the levels of histone acetylation, as determined by the ratio of acetylated H3 per total histone H3, were not significantly altered upon stress in cells lacking Sin3/Elp3 compared to wild-type cells (Figure 1E for *gpd1* and Figure S1A for *ctt1*). Also, a similar decrease in total histone H3 levels at CESR genes, as an indicator of nucleosome eviction, was detected upon stress imposition in both wild-type and *Δsin3/elp3* cells (Figure 1F for *gpd1* and Figure S1B for *ctt1*). Thus, we conclude that stress-dependent histone acetylation and nucleosome eviction at stress genes does not significantly rely on Sin3/Elp3.

### Sin3/Elp3 is required to modify the tRNAs of Lys, Glu and Gln at their wobble U

**Figure 2:**
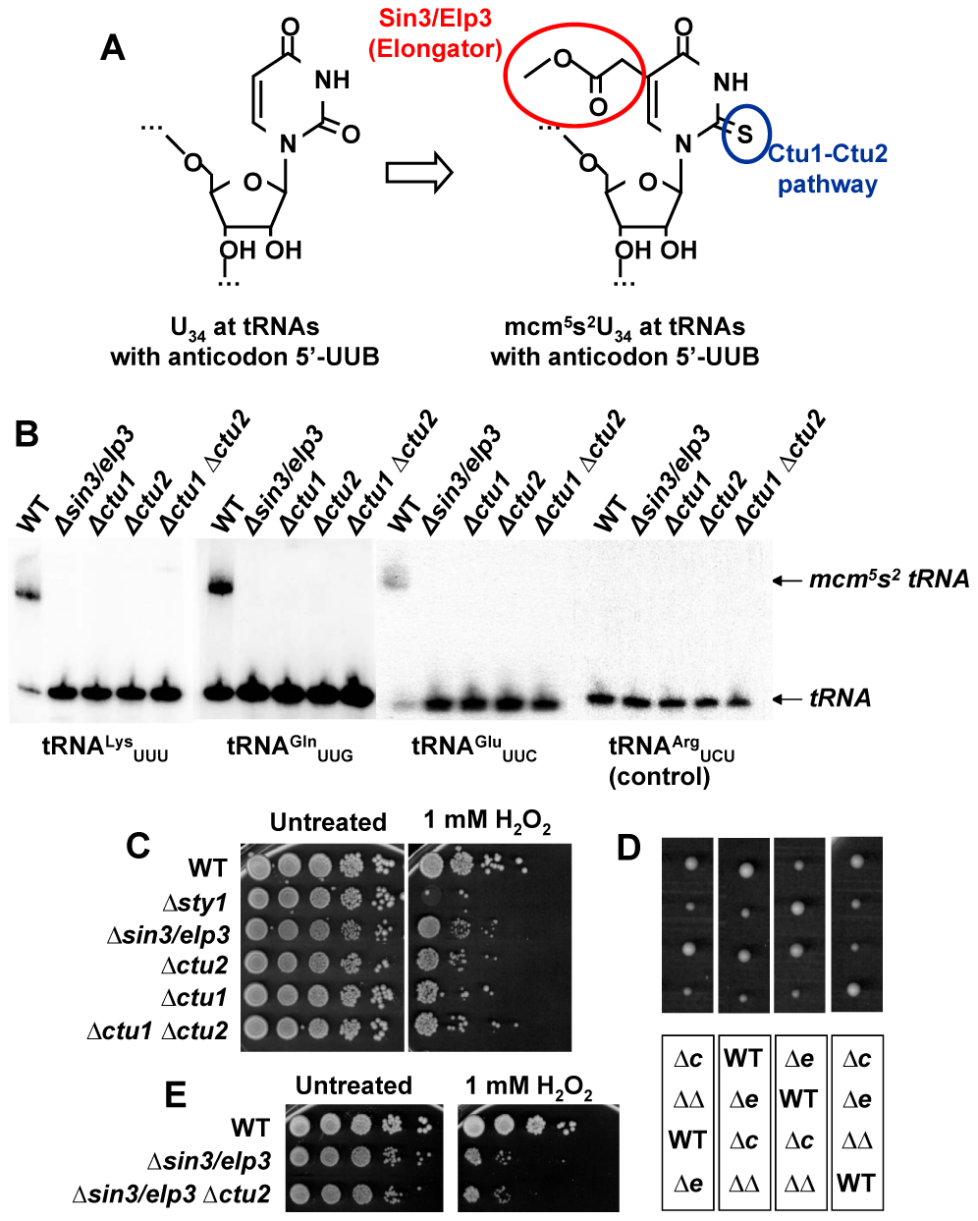
Sin3/Elp3 is required for modification of uridine-34 (U_34_) at the anticodon of some cytoplasmic tRNAs. (A) Sin3/Elp3 and the Ctu1-Ctu2 pathways are required for the mcm^5^ and s^2^ modifications, respectively, at U_34_ of some anticodons. (B) Northern blot analysis of bulk tRNA isolated from WT (972), IV16 (*Δsin3/elp3*), YDH 644 (*Δctu1*), IV86 (*Δctu2*), and YDH 254 (*Δctu1 Δctu2*) using specific probes against tRNA^Lys^_UUU_, tRNA^Gln^_UUG_, tRNA^Glu^_UUC_ and tRNA^Arg^_UCU_ (negative control) by the APM-gel retardation method. The position of the unmodified (*tRNA*) or modified (*mcm^5^s^2^ tRNA*) tRNAs are indicated with arrows. (C) *Δctu1* and *Δctu2* strains are sensitive to oxidative stress. Serial dilutions from cultures of strains 972 (WT), AV18 (*Δsty1*), IV16 (*Δsin3/elp3*), IV86 (*Δctu2*), YDH 644 (*Δctu1*), YDH 254 (*Δctu1 Δctu2*) were spotted onto rich plates without (Untreated) or with 1 mM H_2_O_2_. (D) Double mutant *Δsin3*/*elp3 Δctu2* colonies are similar in size to single mutant *Δsin3*/*elp3*. Tetrad analysis of a cross between IV16 (*Δsin3/elp3*) and IV86 (*Δctu2*) strains. Each vertical box corresponds to the four spores of a tetrad. The genotypes of each colony of the top panel are indicated in the corresponding positions of the bottom panel: WT, *Δctu2 (Δc), Δsin3/elp3 (Δe) or Δsin3/elp3 Δctu2 (ΔΔ)*. (E) Single mutant *Δsin3*/*elp3* and double mutant *Δsin3*/*elp3 Δctu2* show similar sensitivity to oxidative stress. Serial dilutions from cultures of strains 972 (WT), IV16 (*Δsin3/elp3*) and JF73 (*Δsin3/elp3 Δctu2*) were spotted onto rich plates without (Untreated) or with 1 mM H_2_O_2_.

As detailed in the Introduction, some cytoplasmic tRNAs (those with a UUB anticodon, ‘B’ being U, C or G in the tRNAs for Lys, Glu of Gln, respectively), are subjected to diverse modifications at their U_34_ (5′ position) of the anticodon to yield mcm^5^s^2^U_34_ (Figure 2A). Thus, Elongator mutants in *S. cerevisiae* have been shown to fully lack the mcm^5^U modification [12], and present a 50% decrease in the s^2^U modification at these specific tRNAs [27], [30]. In an attempt to confirm the requirement of Sin3/Elp3 in the dual modification of these tRNAs, we purified tRNA and used an electrophoretic mobility shift assay which allows the detection of thio-containing tRNA molecules [37]. As a control, we used strains lacking Ctu1 and Ctu2, which had recently been demonstrated to be required for the thiolation step [33] (Figure 2A). As we show in Figure 2B, Sin3/Elp3 seems to be also required for thiolation of U_34_ at tRNA^Lys^_UUU_, tRNA^Gln^_UUG_ and tRNA^Glu^_UUC_. The presence of a contaminant inhibitor in the tRNA samples of the different mutants was discarded by mixing those with wild-type RNA and performing the same band shift assay: the thio-contaning tRNA molecules of wild-type RNA were perfectly detected (Figure S2). These findings suggest that introduction of the mcm^5^ at U_34_ is the first step in the dual modification at these tRNA anticodon residues in *S. pombe*. This is consistent with the observation in budding yeast that defects in Elongator not only abolish the mcm^5^ modification, but also partially compromise the thiolation of position 2 of U [27], [30], whereas the absence of the Ctu2 homologue, Ncs2, does not affect the formation of the mcm^5^ chain [15].

### Defects in U_34_ thiolation by Ctu1-Ctu2 are also rendering cells sensitive to oxidative stress

According to our results, Sin3/Elp3 is required for the mcm^5^s^2^U_34_ modification of tRNAs but does not affect histone H3 acetylation. This suggests that the absence of the mcm^5^s^2^U_34_ modification could be the cause of stress sensitivity of cells lacking Sin3/Elp3. If this hypothesis is correct, then *Δctu1* or *Δctu2* strains should share the same phenotypes as Elongator mutants, as shown before in budding yeast [15]. As shown in Figure 2C, the tolerance to peroxides of strains lacking Sin3/Elp3, Ctu1 or Ctu2 is very similar and lower than that of wild-type cells. Furthermore, cells lacking Sin3/Elp3 and Ctu2 are not synthetic lethal (Figure 2D) and the double mutant displays similar H_2_O_2_ sensitivity than the *Δsin3/elp3* mutant (Figure 2E), suggesting that the catalytic activities of the Elongator and Ctu1/2 complexes are sequential (Figure 2B), and that the absence of only one of them is sufficient to avoid full modification of the wobble U_34_ nucleosides and disturb the function of the target tRNAs.

### The stress phenotypes of cells lacking Sin3/Elp3 or Ctu1-Ctu2 are suppressed by over expression of tRNA^Lys^_UUU_

**Figure 3:**
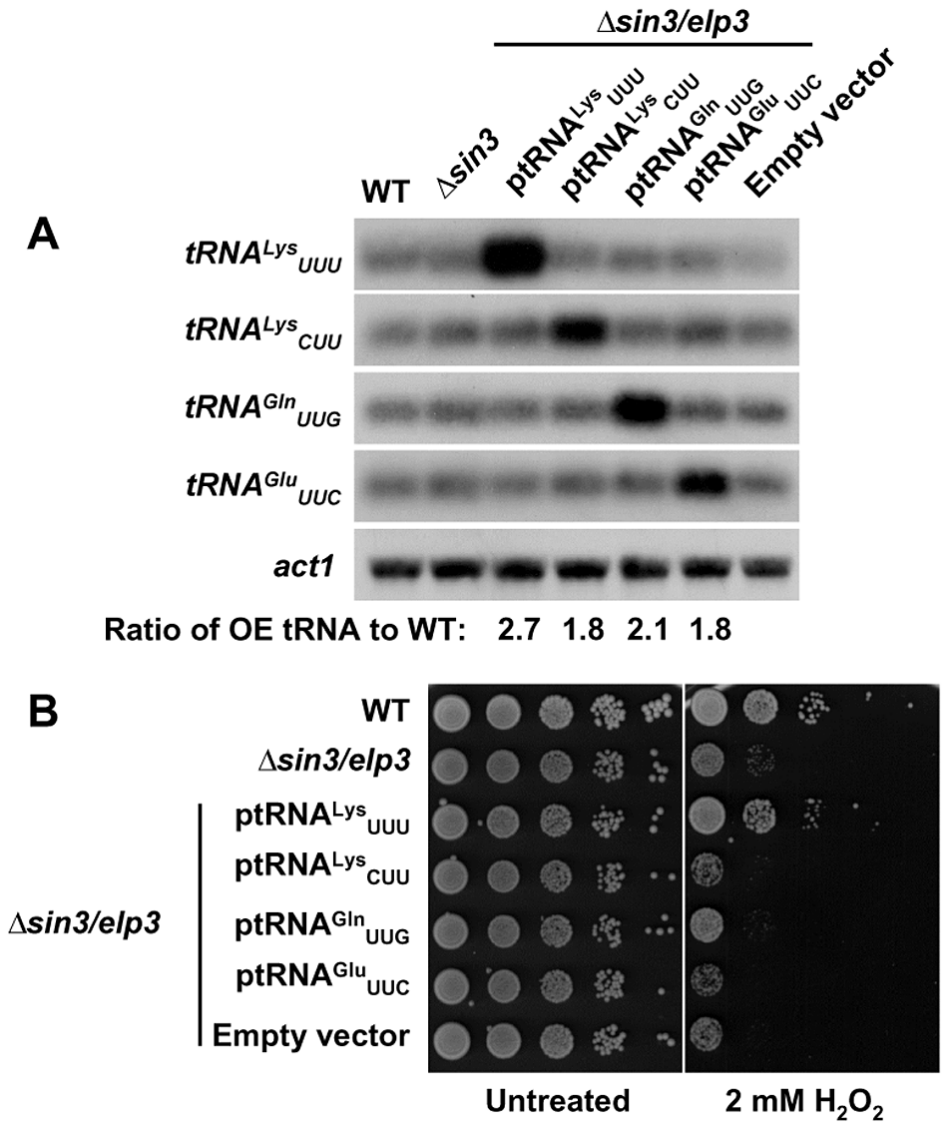
Over-expression of tRNA^Lys^_UUU_ supresses the growth defects of *Δsin3/elp3* upon oxidative stress. (A) Relative levels of tRNA over-expression by Northern blot. Total RNA from strains 972 (WT), IV16 (*Δsin3/elp3*), or JF77 (*Δsin3/elp3*) transformed with episomal plasmids p465 (ptRNA^Lys^_UUU_), p466 (ptRNA^Lys^_CUU_), p467 (ptRNA^Gln^_UUG_), p468 (ptRNA^Glu^_UUC_) or the empty vector pREP.42x, was analyzed by Northern blot with probes of dsDNA of the indicated tRNAs labeled at their antisense strand. a*ct1* is shown as a loading control. The numbers below last panel indicate the relative levels of the corresponding over-expressed tRNA normalized to basal levels in wild-type strain. (B) Serial dilutions from cultures of strains as in A were spotted onto synthetic rich media plates without (Untreated) or with 2 mM H_2_O_2_.

Cells lacking Sin3/Elp3, Ctu1 or Ctu2 cannot generate the mcm^5^s^2^U_34_ modification at some specific tRNAs, and are sensitive to oxidative stress. These modifications have been proposed to contribute to either translation efficiency [22]–[25] or fidelity [19]–[21] of mRNAs containing the complementary codons. If the stress sensitivity is a consequence of defects in codon specific translation, then over-expression of the target tRNAs may alleviate the phenotypes, as shown before in *S. cerevisiae* [15]. We constructed episomal plasmids and used them to over-express different tRNAs in *Δsin3/elp3* cells (Figure 3A). As shown in Figure 3B, the sensitivity to peroxides of cells lacking Sin3/Elp3 was largely rescued with over-expression of tRNA^Lys^_UUU_, but not with plasmids containing other tRNAs which are also modified by Elongator and by Ctu1-Ctu2, such as those for tRNA^Gln^_UUG_ or tRNA^Glu^_UUC_. As a negative control, complementation was not observed over-expressing the Elongator-independent tRNA^Lys^_CUU_. Sensitivity of cells lacking Ctu2 was also suppressed with tRNA^Lys^_UUU_ over-expression (Figure S3). We conclude that this complementation provides genetic evidence for the role of tRNA modification in the *Δsin3/elp3* phenotype, and in particular for the role of modified tRNA^Lys^_UUU_ in the stress response.

### The codon usage for Lys at stress genes is not optimized for highly expressed genes

Modifications of U_34_ at the tRNAs for Lys, Gln and Glu (with UUB anticodons) are important for proper cell growth, and *S. cerevisiae* cells lacking one or several genes required for these modifications display various phenotypes [15], [26]. However, the function of these modifications in the process of translation is far from being understood. In recent years, mcm^5^s^2^U_34_ tRNA modifications have been proposed to modulate translation through a number of distinct mechanisms: increasing the efficiency of codon recognition [25] or aminoacyl-tRNA synthetase interaction [22]–[24]; or increasing translation fidelity by helping to bind only to the correct codon [19], by avoiding frame shifts [20], or both [21]. Lys, Gln and Glu can be coded by two nucleotide triplets, each of which can be recognized by a specific tRNA; only one of the two tRNAs for each of the three amino acids carries a U at the 5′ position of the anticodon which is modified by the Elongator and Ctu1-Ctu2 complexes (see Table 1). The intracellular concentration of each tRNA is assumed to be proportional to the number of copies of the tRNA coding genes [38]–[41]. Regarding the cytoplasmic tRNAs decoding for Lys, Gln and Glu, the *S. pombe* genome displays the largest disequilibrium in gene copy number for the tRNAs for Lys: there are 3 copies of tRNA^Lys^_UUU_ gene but 9 copies of the tRNA^Lys^_CUU_ gene (Table 1). However, the overall codon usage for Lys in fission yeast indicates that 62% of the Lys codons require the less abundant tRNA^Lys^_UUU_ [42] (Table 1), and this codon usage dramatically changes for highly expressed genes, where only 10% of the codons are read by the Sin3/Elp3 modifiable tRNA^Lys^_UUU_. This bias of codon usage seems to be a result of optimizing translation during evolution, since the abundant tRNAs should be used for the translation of highly expressed mRNAs, which may require an efficient and fast translation machinery [42].

**Table 1:** Codon usage and *tRNA* gene copy number for Lys, Gln and Glu.

		Number of mRNA molecules per cell^d^	Codon usage Lys^e^		Codon usage Gln^e^		Codon usage Glu^e^	
			5′-AAA (3 genes tRNA^Lys^_UUU_)^f^	5′-AAG (9 genes tRNA^Lys^_CUU_)^f^	5′-CAA (4 genes tRNA^Gln^_UUG_)^f^	5′-CAG (2 genes tRNA^Gln^_CUG_)^f^	5′-GAA (4 genes tRNA^Glu^_UUC_)^f^	5′-GAG (6 genes tRNA^Glu^_CUC_)^f^
**Total ** ***S. pombe*** ** ORFs** a	Basal levels	N.P.	0.62	0.38	0.72	0.28	0.68	0.32
**Highly expressed ORFs** b	Basal levels	256.53	0.10	0.90	0.90	0.10	0.35	0.65
**50 H_2_O_2_-up regulated genes** c	Basal levels	5.9	0.55	0.45	0.74	0.26	0.65	0.35
	Induced levels	145.07	0.55	0.45	0.74	0.26	0.65	0.35
***atf1*** ** mRNA**	Basal levels	6.2	0.61	0.39	0.66	0.34	0.74	0.26
	Induced levels	18.2	0.61	0.39	0.66	0.34	0.74	0.26
***pcr1*** ** mRNA**	Basal levels	8.0	0.67	0.33	0.52	0.48	0.44	0.56
	Induced levels	54.8	0.67	0.33	0.52	0.48	0.44	0.56

N.P: Not provided.

a 4932 *S. pombe* ORFs are described in [42].

b 30 ORFs with highest expression levels, obtained from [42].

c 50 genes most up-regulated after treatment with 0.5 mM of H_2_O_2_ during 30 minutes [4].

d The number of mRNA molecules per cell at basal levels was obtained from [42].

The number of mRNA molecules per cell at induced levels was calculated using the fold induction ratios upon 0.5 mM of H_2_O_2_ during 30 minutes from microarray experiments reported at [4].

e Codon usage values of ‘Total *S. pombe* ORFs’ and ‘Highly expressed ORFs’ were obtained from [42].

The other codon usage values were calculated using free software ACUA v1.0 [43].

f tRNA gene copy number was extracted from PomBase [61].

The CESR genes are highly expressed upon stress conditions. We wanted to test whether upon induction these stress genes reach mRNA levels which are comparable to those of highly expressed genes. Using microarray expression data from basal [42] and H_2_O_2_ conditions [4], we calculated the mean number of mRNA molecules per cell for the 50 most induced genes upon peroxide exposure (Table 1). On average, we calculated 145 mRNA molecules per cell, which is comparable to the mean 257 mRNA molecules per cell for the group of ‘Highly expressed ORFs’ (open reading frames) [42]. We further calculated the codon usage for these subset of *S. pombe* genes using the ACUA software [43], and found it similar to the mean of total fission yeast genes (Table 1). This indicates that there has not been an evolutionary adaptation to change the codon usage of the stress genes, which are only highly expressed under some stress conditions. Instead, optimization of the recognition of the Lys codon AAA by the less abundant tRNA^Lys^_UUU_ at H_2_O_2_-up regulated mRNAs seems to be achieved by modifying the wobble nucleoside U_34_. That explains why the U_34_-modifying activities are specifically critical especially for stress survival.

### The stress-dependent protein levels, but not the mRNA levels, of the transcription regulators Atf1 and Pcr1 are severely decreased in cells lacking Sin3/Elp3

**Figure 4:**
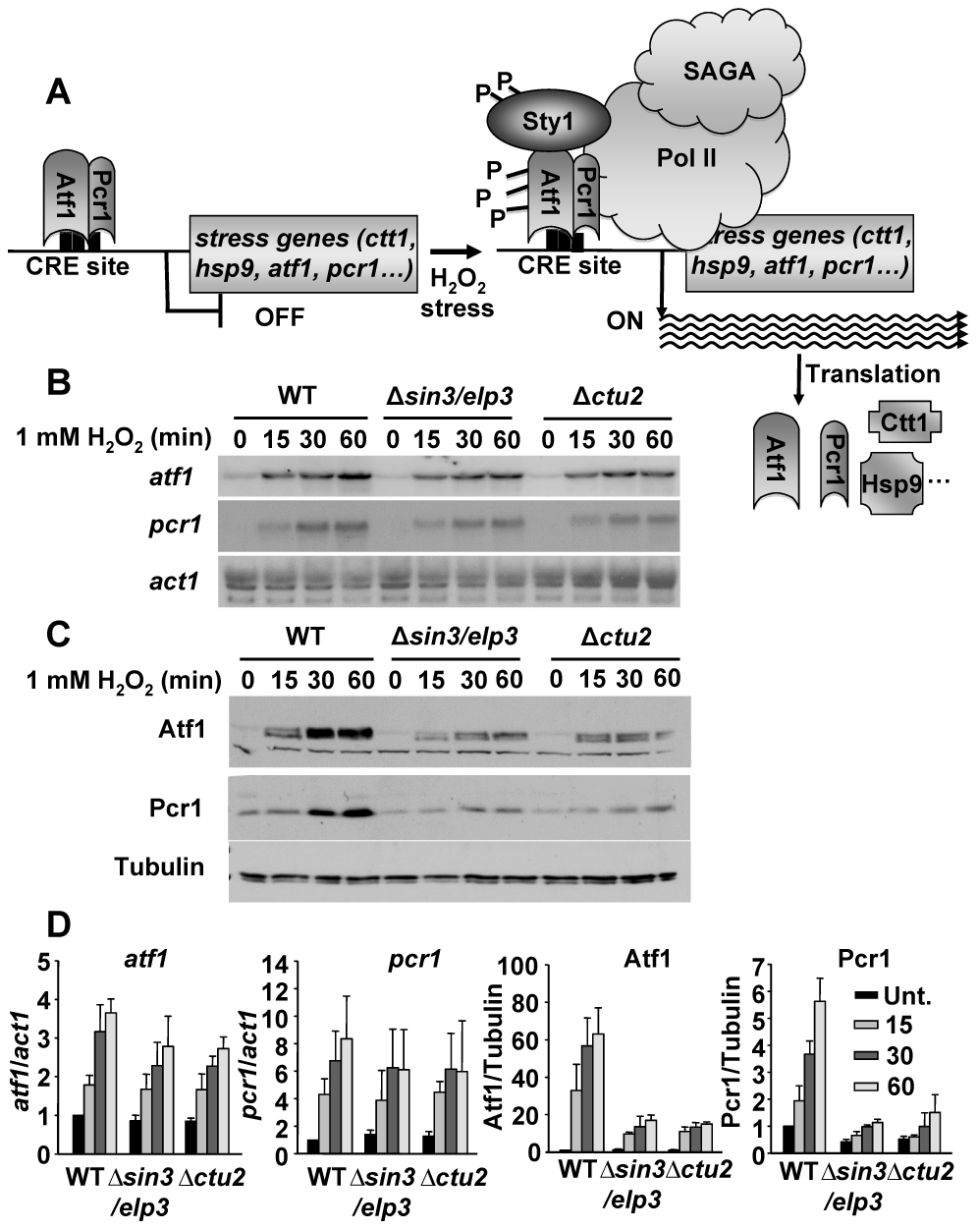
Protein levels of the stress transcription factors Atf1 and Pcr1 depend on the U_34_ modifying activities Sin3/Elp3 and Ctu2. (A) Scheme illustrating the activation of the stress gene expression program by Atf1 and Pcr1 (see text for details). (B and C) Absence of Sin3/Elp3 or Ctu2 barely affects transcription of the *atf1* and *pcr1* genes but largely affects Atf1 and Pcr1 protein levels. Rich media cultures of strains 972 (WT), IV16 (Δ*sin3/elp3*) and IV86 (Δ*ctu2*) treated with 1 mM H_2_O_2_ at the indicated time points were analyzed to determine transcription levels of *atf1* and *pcr1* genes by Northern blot (B), or Atf1 and Pcr1 protein levels by Western blot using polyclonal antibodies (C). *act1* mRNA or tubulin were used as loading controls for B and C, respectively. (D) Quantification of the relative mRNA and protein levels for *atf1*, *pcr1*, Atf1 and Pcr1 in wild-type and mutant strains. The Northern or Western blot panels of experiments as in B and C, respectively, were quantified and represented here relative to untreated wild-type levels (with an assigned value of 1). The *atf1* and *pcr1* mRNA levels normalized to *act1* are shown in the left two panels, *w*hereas the Atf1 and Pcr1 protein levels normalized to tubulin are shown in the two right panels. Error bars (SEM) were calculated from biological duplicates.

If lack of Sin3/Elp3 is causing defects due to its tRNA-modifying activity, then translation of stress mRNAs may be affected. The transcriptional response to stress is driven by the MAP kinase Sty1 and the heterodimeric transcription factor Atf1-Pcr1, which trigger massive transcriptional changes. Based on our analysis of codon usage we speculated that induction of CESR gene expression should be accompanied with efficient translation of the newly synthesized mRNAs (Figure 4A). We used *act1* mRNA and tubulin as loading and quantification controls in our Northern and Western blots, respectively. Whereas the gene coding for actin is constitutively expressed, the *atf1* and *pcr1* genes are induced upon H_2_O_2_ exposure (Figure 4B; WT), and proper over-expression of Atf1 upon stress is required to fully achieve a complete transcriptional cellular response. The codon usage of the genes coding for these transcriptional regulators has not been evolutionary adapted towards highly expressed genes (Table 1). We therefore speculated that Atf1 and Pcr1 protein synthesis, rather than their mRNA accumulation, would be defective in cells missing the tRNA^Lys^_UUU_-modifying activities Sin3/Elp3 or Ctu2, as would occur with the expression of all the other CESR proteins. As shown in Figure 4B, H_2_O_2_-dependent expression of *atf1* and *pcr1* mRNAs is not dramatically impaired in cells lacking Sin3/Elp3 or Ctu2. However, the amount of translated Atf1 and Pcr1 proteins are clearly diminished in the mutant strains (Figure 4C). Thus, the defective accumulation upon stress of the Atf1-Pcr1 transcription factor and other CESR proteins in the *Δsin3/elp3* or *Δctu2* strains (Figure 4D) could explain the sensitivity to stress of cells lacking any of these two tRNA-modifying complexes. We also determined that moderate levels of over-expression of tRNA^Lys^_UUU_ (Figure 3A) were not able to fully recover the wild-type levels of Atf1 or Pcr1 proteins in the mutant strains, as determined by Western blot (data not shown): we suspect that since up to 500 stress genes are expressed more than 2-fold upon 0.5 mM H_2_O_2_ stress [4], most of the corresponding mRNAs may need excess tRNA^Lys^_UUU_ for proper translation in *Δsin3/elp3* or *Δctu2* strains.

**Figure 5:**
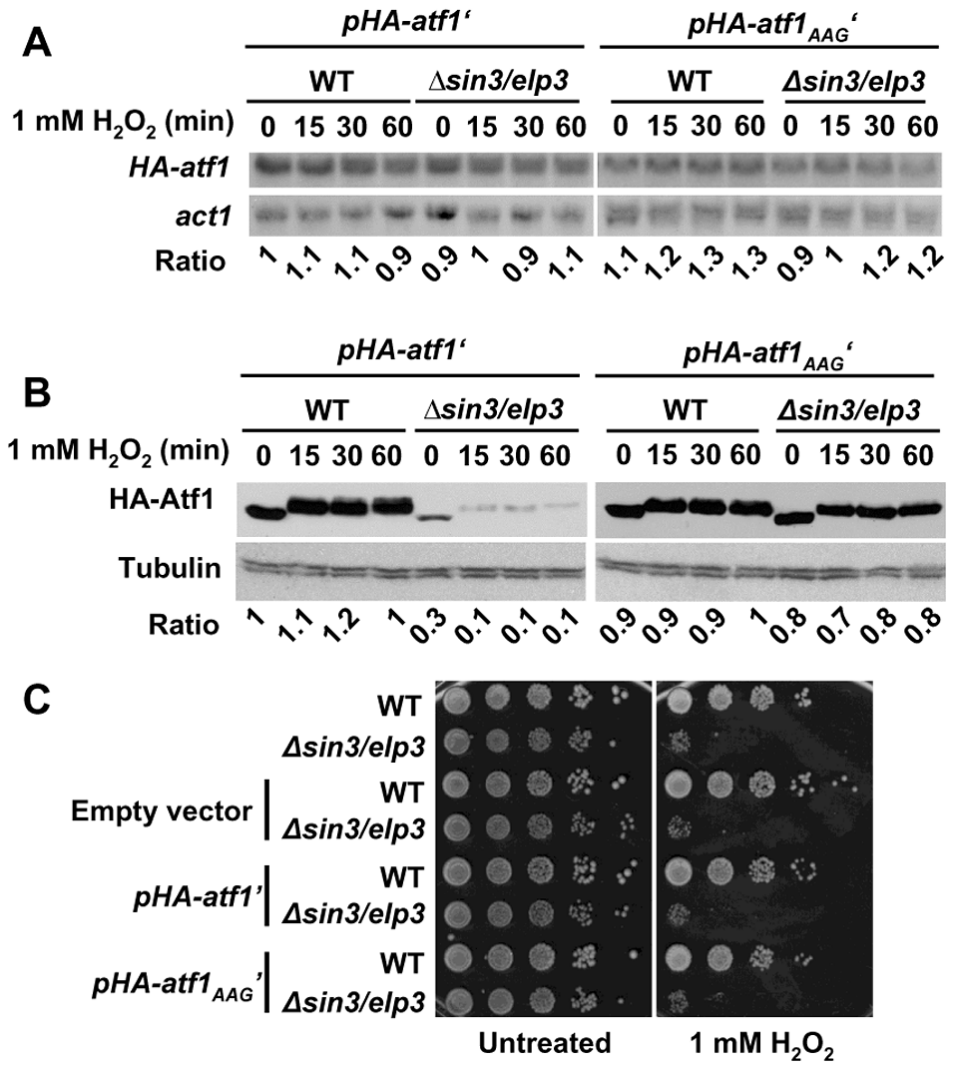
Expression of a synthetic AAA-to-AAG *atf1* gene rendered wild-type Atf1 protein levels in Elongator mutants. (A and B) Vectors carrying a constitutively expressed wild-type (p*HA-atf1*′) or mutated *atf1* gene (p*HA-atf1*_AAG_′) were integrated in the chromosomes of wild-type or *Δsin3/elp3* strains. Rich media cultures of strains JF91 (WT+p*HA-atf1*′), JF92 (*Δsin3/elp3*+p*HA-atf1*′), JF94 (WT+p*HA-atf1*_AAG_′) and JF95 (*Δsin3/elp3*+p*HA-atf1*_AAG_′), either untreated (0) or treated with 1 mM H_2_O_2_ for the indicated times, were analyzed to determine *HA-atf1* mRNA levels by Northern blot using an anti-HA probe (A) or HA-Atf1 protein levels by Western blot using monoclonal antibody against HA (B). The numbers below the Northern or Western blot panels indicate the relative levels of *HA-atf1/act1* mRNAs (panel A) or HA-Atf1/tubulin protein levels (panel B). (C) Expression of the mutant AAA-to-AAG Atf1 protein does not suppress the growth defects of *Δsin3/elp3* cells upon oxidative stress. Empty vector or plasmids carrying a wild-type (p*HA-atf1*′) or a mutated *atf1* gene (p*HA-atf1*_AAG_′) were integrated in the chromosomes of wild-type or *Δsin3/elp3* strains. Cultures from the resulting strains JF88 (WT+empty vector), JF89 (*Δsin3/elp3*+empty vector), JF91 (WT+p*HA-atf1*′), JF92 (*Δsin3/elp3*+p*HA-atf1*′), JF94 (WT+p*HA-atf1*_AAG_′) and JF95 (*Δsin3/elp3*+p*HA-atf1*_AAG_′) were serially diluted and spotted onto rich media plates without (Untreated) or with 1 mM H_2_O_2_.

Further confirmation of the role of Sin3/Elp3 on mRNA translation came from the fact that expression of a synthetic *atf1* gene, in which all the AAA codons of its ORF had been changed by the synonymous AAG, rendered Atf1 expression not sensitive to the absence of Sin3/Elp3 or Ctu2 (Figure 5 and Figure S4 for wild-type and AAA-to-AAG HA-Atf1 expressed from an heterologous and constitutive promoter; Figure S5 for wild-type and AAA-to-AAG Atf1 expressed from its own promoter). The shift in electrophoretic mobility of the H_2_O_2_- and Sty1-dependent phosphorylated Atf1 has been widely reported [44], [45]. Importantly enough, cells lacking Sin3/Elp3 or Ctu2 expressing the AAG-only Atf1 and therefore reaching wild-type levels of Atf1 still displayed sensitivity to grow on H_2_O_2_ plates (Figure 5C for *Δsin3/elp3* and Figure S4C for *Δctu2*), since the Lys codon usage of the hundreds of genes over-expressed upon oxidative stress remain rich in the AAA codon (Table 1).

## Discussion

Optimal performance of a biological process such as cellular adaptation to environmental changes requires that a complex phenomenon like protein expression to be carried out with high efficiency and fidelity. Thus, not only transcription but also mRNA homeostasis and translation have to be performed with maximum efficiency, or survival would be hampered. Our study not only provides new insights into the way cells respond to oxidative stress, but also provides important clues regarding the role of the Elongator complex in translation efficiency. Furthermore, our study reveals how the absence of the mcm^5^s^2^U_34_ modification at tRNA^Lys^_UUU_ in cells lacking Sin3/Elp3 or Ctu1/Ctu2 contributes to the observed phenotype of sensitivity to peroxides of these mutant strains.

One of the singularities of the redundancy of the genetic code is that it allows to choose between alternative codons for the same amino acid, and that may exert important consequences on the efficiency of translation depending mainly on the concentrations of each one of the corresponding tRNAs. As intracellular concentrations of different tRNAs are not easily measured, the amount of each species in cells are often considered to be proportional to the copy number of the tRNA-coding genes in the genome [38], [41]. Thus, codon usage may be biased towards the use of abundant tRNAs when strong and efficient translation is required, i.e. highly expressed genes. Out of the three tRNAs species modified at their 5′-uridine of the anticodon to mcm^5^s^2^U_34_, tRNAs for Lys display the largest imbalance according the gene copy number (only one tRNA^Lys^_UUU_ for 3 tRNA^Lys^_CUU_; Table 1). Accordingly, the codon usage for highly expressed ORFs suffers the major deviation from the average *S. pombe* ORFs for the Lys codons (only 10% of the Lys codons are AAA in highly expressed ORFs, whereas the average is 62%; Table 1). This adaptation of the Lys codon usage in highly expressed genes, which has not been adopted by the CESR genes, would justify the need for the mcm^5^s^2^U_34_ modification at the tRNA^Lys^_UUU_ to set up a strong stress response, which should enhance the efficiency of translation of the *atf1* and *pcr1* mRNAs.

Recently, a mass spectrometry-based analysis by the groups of Dedon and Begley has revealed that the spectrum of nucleoside modifications of *S. cerevisiae* changes upon cell exposure to H_2_O_2_ and other stressors [46]. In some cases, but not all, increases of a specific modified ribonucleoside correlate with stress sensitivity of mutant cells lacking an enzyme involved in its biosynthesis [46]. In particular, the mcm^5^s^2^U_34_ is not enhanced by peroxides in budding yeast, while other modifications such as m^5^C are [47]. Similarly, cells lacking Trm9, required to introduce the mcm^5^s^2^U_34_ in yeast, are not sensitive to H_2_O_2_ [46], while we have shown here that cells lacking Elongator components, or Ctu1 or Ctu2, display impaired survival when exposed to peroxides. How the changes in modified nucleosides are triggered upon stress in *S. cerevisiae* is still to be determined. Whether the mcm^5^s^2^U_34_ modification accumulates upon H_2_O_2_ in *S. pombe* will have to be elucidated, but at least the activities required for this modification (Elongator and Ctu1-2) are not up-regulated at the transcriptional level upon peroxide exposure [4].

We have shown here that generation of the mcm^5^U_34_ by Elongator is required for wild-type tolerance to oxidative stress. In the absence of Elongator, the thiolation at carbon 2 of U_34_ is not generated by Ctu1-Ctu2 (Figure 2A), since cells lacking Sin3/Elp3 do not incorporate any modification (mcm^5^ or S^2^) at U_34_. However, cells lacking Ctu2 or Ctu1 accumulate mcm^5^U_34_ [33]; unfortunately, this modification does not seem to be sufficient to allow proper translation of *atf1* and *pcr1* mRNAs (Figure 4B), and therefore *Δctu2* cells show sensitivity to peroxides (Figure 2C).

Cells lacking Sin3/Elp3 already display some defects in the absence of stress, since translation of some growth-related mRNAs is probably dampened as well. Thus, the size of colonies derived from *Δsin3/elp3* spores is significantly smaller than that of wild-type or *Δctu2* ones (Figure 2D). Also, both the duplication time and maximum OD_600_ of cultures are defective in cells lacking Sin3/Elp3 (Figure S6A). Importantly enough, the growth-related *Δsin3/elp3* defects are totally suppressed by over-expression of tRNA^Lys^_UUU_ (Figure S6B). It has recently been reported that fission yeast cells devoid of Elongator result in three phenotypes unrelated to stress: thermosensitivity, cell elongation and multiple and misplaced septa, common to defects in cell-cycle progression [11], [34]. In this report, the authors performed a screen of the fission yeast proteome for translational defects which pointed towards the kinase Cdr2, a central regulator of mitosis, as the target of translational control by Elongator due to an unusual Lys codon usage bias of its mRNA. Analysis of their proteome-wide data indicates that Atf1 and Pcr1 are also down-regulated even under basal, unstressed conditions, supporting the conclusions of our manuscript [34]. It is worth pointing out that cells lacking Ctu2 display defects in *atf1* and *pcr1* translation and subsequent sensitivity to stress, whereas they do not seem to have growth defects (Figure 2D and Figure S6A). This suggests that the non-thiolated mcm^5^U_34_ tRNAs of *Δctu2* cells may have some intermediate activity, indicating that some mRNAs could be efficiently translated but not others.

In conclusion, our study points to the fact that the biologically essential functions of Elongator in *S. pombe* are related to tRNA modification. This function is significantly relevant for the efficient translation of mRNAs with a biased high AAA to AAG ratio of codons for Lys, especially when these mRNAs are expressed at high copy number. Highly expressed house-keeping genes have evolutionary circumvented this problem by enhancing the AAG to AAA ratio. The wobble position U_34_ of tRNAs with an UUB anticodon is almost universally modified [48]–[50], and our report suggests that specific highly expressed mRNAs with a high VAA content (‘V’ being A, C or G) should be inefficiently translated in the absence of Elongator, what would determine a particular phenotype for each cell type.

## Materials and Methods

### Yeast strains, plasmids and growth conditions

We used the wild-type *S. pombe* 972 (*h^−^*) and mutants thereof. The origins and genotypes of strains used in this study are outlined in Table S2. To construct episomal plasmids containing tRNA coding genes, the tRNA genomic sequences were PCR-amplified from *S. pombe* genomic DNA using primers specific for the tRNAs' upstream and downstream sequences, and cloned into the fission yeast episomal plasmid pREP.42x [51], with a *ura4*-selectable marker, previously digested with *PstI* and *SacI* to eliminate the *nmt* promoter and the transcription terminator sequences, so that each tRNA is expressed from its endogenous sequences (500 bp genomic flanking sequences on each side of the tRNAs). The replication origin *ars1* contained in the pREP.42x vector allows an average plasmid copy number of approximately 8 copies/cell [52]. We obtained plasmids p465 [expressing tRNA^Lys^_UUU_ (SPBTRNALYS.06)], p466 [expressing tRNA^Lys^_CUU_ (SPCTRNALYS.11)], p467 [expressing tRNA^Gln^_UUG_ (SPBTRNAGLN.02)] and p468 [expressing tRNA^Glu^_UUC_ (SPATRNAGLU.02)]. All the PCR-amplified DNA fragments cloned in these plasmids were confirmed by sequencing. To generate a mutated version of *atf1* where all eleven AAA codons were replaced by AAG, full length gene synthesis was performed (GeneScript). The inducible *nmt* promoter of plasmid p123.41x [53] was replaced by the constitutive *sty1* promoter (0.8 kb from ATG). The resulting plasmid was digested to release the *sty1* promoter fused to the *HA* coding sequence, and cloned into pAY025 [54] to obtain the plasmid p386′ (empty vector). Then, the *atf1* and *atf1_AAG_* ORFs were cloned into p386′ to obtain p428′ and p428′_AAG_ respectively. These plasmids, allowing the constitutive expression of HA-Atf1 and HA-Atf1_AAG_, were integrated at the *leu1* loci of wild-type and mutant strains, yielding strains JF91 to JF96 (see Table S2). Both wild-type and mutated *atf1*_AAG_ alleles were also introduced at the endogenous *atf1* locus of strain JF85, JF86 (*Δsin3/elp3*) and JF87 (*Δctu2*) by standard recombination techniques, yielding strains JF106, JF107 and JF108 (wild-type *atf1*) and JF109, JF110 and JF111 (*atf1*_AAG_) (see Table S2). Cells were grown in rich medium (YE) in most of the experiments, or in synthetic minimal medium when indicated [55].

### H_2_O_2_ sensitivity assay

For survival on solid plates, *S. pombe* strains were grown, diluted and spotted (10^5^ to 10 cells per spot) in YE medium or synthetic minimal medium agar plates as described previously [54], containing or not H_2_O_2_ at the indicated concentrations.

### RNA analysis

Total RNA from *S. pombe* rich medium cultures was obtained, processed and transferred to membranes as described previously [56]. Membranes were hybridized with the [α-^32^P]dCTP-labelled *ctt1, gpd1, hsp9, srx1, gpx1, atf1, pcr1* and *act1* probes, containing the complete ORFs. To determine the levels of tRNA over-expression in strains carrying episomal tRNA plasmids, we PCR-amplified from genomic DNA 300-bp products corresponding to each specific tRNA, and then performed a primer extension of each antisense strand with Klenow polymerase, dNTPs and [α-^32^P]dCTP, following standard molecular biology techniques [57]. A similar strategy was used to label the HA-coding DNA, which was used in Figures 5A and S4A. Membranes were exposed to a phosphorimager plate (GE Healthcare) and scanned on a Typhoon 8600 (GE Healthcare). Relative quantifications were performed using the ImageQuant 5.2 program (GE healthcare), using *act1* mRNA as loading control.

### Preparation of *S. pombe* TCA extracts and immuno blot analysis

Modified trichloroacetic acid (TCA) extracts were prepared as previously described [54]. Immunoblotting to analyze the *in vivo* acetylation state of total histone H3 was performed as described previously [7]. Pcr1 and Atf1 were immunodetected with polyclonal anti-Pcr1 and polyclonal anti-Atf1 antiserums, as described previously [54]. HA-Atf1 was detected with monoclonal anti-HA antibody. Monoclonal anti-tubulin (Sigma) was used as a loading control. Relative quantifications of protein levels in Western blots was performed using the free Image J software.

### Chromatin immunoprecipitation

To test histone H3 acetylation and total H3 upon stress imposition, the indicated strains were grown in rich media, and chromatin isolation and immunoprecipitation was performed as described previously [7]. The error bars (SEM) were calculated from biological triplicates.

### tRNA isolation

Cells were grown at 30°C in 100 ml rich media and harvested at OD_600_ of 0.5. The cell pellet was resuspended in 4 ml 0.9% NaCl. The cells suspension was vortexed at room temperature for 5 min in the presence of 4 ml of acidic phenol and 3 ml of glass beads. Subsequently 0.4 ml chloroform were added and the suspension vortexed for another 30 sec. The suspension was cleared by centrifugation at 3000 rpm for 20 min at room temperature. The water phase was collected and re-extracted with 2 ml of acidic phenol and 0.2 ml of chloroform until the interphase was clean. The final water phase was collected, mixed with 2.5 vol of 100% ethanol and 0.1 vol of 20% potassium acetate to precipitate tRNA. Precipitated tRNA was purified by column purification as described previously [58].

### tRNA modification analysis

0.5 µg of bulk tRNA per lane (or mixed 0.5 µg+0.5 µg of two different types of bulk tRNA per lane when indicated; Figure S2) were analyzed on 10% acrylamide gels, 0.5× TBE; 7 M urea. (*N*-Acryloylamino) phenyl mercuric chloride (APM) was added to a final concentration of 50 µg per ml. Northern blot analysis was performed essentially as described previously [59], using probes CTCCCACTGCGAGATTCGAACTCGC to detect tRNA^Lys^_UUU_, GGTCGTACTGGGAATCGAACCCAGG to detect tRNA^Gln^_UUG_, CTCCGTTGCGGGGAGTCGAA to detect tRNA^Glu^_UUC_ and CTCCCGGCGGGACTCGAA to detect the negative control tRNA^Arg^_UCU_. Membranes were exposed to a phosphorimager plate (GE Healthcare) and scanned on a Typhoon FLA 7000 (GE Healthcare). In the absence of APM in the gels, the corresponding shifts of thiolated tRNAs were not observed (data not shown).

### Tetrad analysis

Tetrad analysis was performed essentially as described [60]. Briefly, asci and spores was separated using the MSM 400 Yeast Dissection Microscope (Singer Instruments) and germinated in YE medium agar plates. To determine the genotype of each spore, colonies generated were replicated into the same media containing or not kanamycin (KAN) and/or nourseothricin (NAT).

## Supporting Information

Figure S1 Sin3/Elp3 does not affect histone modification at CESR genes. (A) Stress-dependent H3 acetylation at CESR genes does not require Sin3/Elp3. Cultures of strains 972 (WT) and IV16 (*Δsin3/elp3*) were treated (+) or not (−) with 1 mM H_2_O_2_ for 5 min. ChIP assays were performed using antibodies specific for acetylated Lys9 and Lys14 of histone H3 (H3Ac) or against unmodified C-terminal domain of H3 (H3). The percentage of immuno precipitation of acetylated H3 versus total H3 is indicated (% IP H3Ac/H3). ChIP experiments were performed using primers covering promoter (prom), coding (ORF) and termination (term) sequences of the *ctt1* gene. (B) Stress-dependent nucleosome eviction at CESR genes does not require Sin3/Elp3. The same experiment as in A is represented here as the percentage of immuno precipitation of total H3 (% IP total H3). Error bars (SEM) for all ChIP experiments were calculated from biological triplicates.
(PDF)

Figure S2 **The bulk tRNA samples of Elongator and Ctu1-Ctu2 mutants do not contain a contaminant inhibitor of tRNA thiolation.** Bulk tRNA isolated from strains WT (972), IV16 (*Δsin3/elp3*), YDH 644 (*Δctu1*), IV86 (*Δctu2*), and YDH 254 (*Δctu1 Δctu2*), were mixed or not as indicated at the top of the panels and analyzed by Northern blot using specific probes against tRNA^Lys^_UUU_, tRNA^Gln^_UUG_, and tRNA^Glu^_UUC_ by the APM-gel retardation method. The position of the unmodified (*tRNA*) or modified (*mcm^5^s^2^ tRNA*) tRNAs is indicated with arrows.
(PDF)

Figure S3 Over-expression of tRNA^Lys^_UUU_ partially supresses the growth defects of *Δctu2* upon oxidative stress. Strain JF78 (*Δctu2*) was transformed with episomal plasmids p465 (tRNA^Lys^_UUU_), p466 (ptRNA^Lys^_CUU_), p467 (ptRNA^Gln^_UUG_), p468 (ptRNA^Glu^_UUC_), or the empty vector pREP.42x. Serial dilutions from cultures of strains 972 (WT), IV86 (*Δctu2*), and JF78 (*Δctu2*) transformed with the indicated plasmids were spotted onto rich media plates without (Untreated) or with 1 mM H_2_O_2_.
(PDF)

Figure S4 Expression of a synthetic AAA-to-AAG *atf1* gene rendered wild-type Atf1 protein levels in Δ*ctu2* mutants. (A and B) Vectors carrying a constitutively expressed wild-type (p*HA-atf1*′) or a mutated *atf1* gene (p*HA-atf1*_AAG_′) were integrated in the chromosomes of wild-type or Δ*ctu2* mutant strains. Rich media cultures of strains JF91 (WT+p*HA-atf1*′), JF93 (Δ*ctu2*+p*HA-atf1*′), JF94 (WT+p*HA-atf1*_AAG_′) and JF96 (Δ*ctu2*+p*HA-atf1*_AAG_′), either untreated (0) or treated with 1 mM H_2_O_2_ for the indicated times, were analyzed to determine *HA-atf1* mRNA levels by Northern blot using an anti-HA probe (A) or HA-Atf1 protein levels by Western blot using monoclonal antibody against HA (B). The numbers below the Northern or Western blot panels indicate the relative levels of *HA-atf1/act1* mRNAs (panel A) or HA-Atf1/tubulin protein levels (panel B), all relative to untreated wild type levels (with an assigned value of 1). (C) Expression of a mutant Atf1 protein does not suppress the growth defects of *Δctu2* upon oxidative stress. Empty vector or plasmids carrying a wild-type (*pHA-atf1*′) or a mutated *atf1* gene (*pHA-atf1_AAG′_*) were integrated in the chromosomes of wild-type or *Δctu2* strains. Cultures from the resulting strains JF88 (WT+empty vector), JF90 (*Δctu2*+empty vector), JF91 (WT+*pHA-atf1*′), JF93 (*Δctu2*+*pHA-atf1*′), JF94 (WT+*pHA-atf1_AAG′_*) and JF96 (*Δctu2*+*pHA-atf1_AAG_*_′_) were serially diluted and spotted onto rich media plates without (Untreated) or with 2 mM H_2_O_2_.
(PDF)

Figure S5 ****Expression of a synthetic AAA-to-AAG *atf1* gene replacing the endogenous *atf1* locus partially recovered wild-type expression levels of Atf1 protein in cells lacking Elongator or Ctu2.**** (A and B) The genomic *atf1* locus of wild-type, Δ*sin3/elp3* or Δ*ctu2* strains was replaced with a mutated *atf1_AAG_* gene. Rich media cultures of strains JF106 (wild type), JF107 (Δ*sin3/elp3*), JF108 (Δ*ctu2*), JF109 (wild type carrying an *atf1_AAG_* allele), JF110 (Δ*sin3/elp3* carrying an *atf1_AAG_* allele), and JF111 (Δ*ctu2* carrying an *atf1_AAG_* allele), either untreated (0) or treated with 1 mM H_2_O_2_ for the indicated times, were analyzed to determine *atf1* mRNA levels by Northern blot (A) or Atf1 protein levels by Western blot using polyclonal antibodies against Atf1 (B). The numbers below the Northern or Western blot panels indicate the relative levels of *atf1/act1* mRNAs (panel A) or Atf1/tubulin proteins (panel B).
(PDF)

Figure S6 **Growth curves of wild-type and tRNA-modifying mutants in minimal medium.** (A) Strains 972 (WT), IV16 (*Δsin3/elp3*), YDH 644 (*Δctu1*) and IV86 (*Δctu2*) were grown in minimal medium and the OD_600_ were recorded at the times indicated. (B) Strains 972 (WT), IV16 (*Δsin3/elp3*) and JF77 transformed with the indicated plasmids (*Δsin3/elp3* tRNA) were grown in minimal medium and the OD_600_ were recorded at the times indicated.
(PDF)

Table S1 **Proteins that constitute the Elongator complex in** *S. cerevisiae*, and their orthologs in *S. pombe*.
(PDF)

Table S2 **Strains used in this study.**
(PDF)
