# Supplementary material for: Modification of tRNALys UUU by Elongator Is Essential for Efficient Translation of Stress mRNAs
Source: PLoS Genet. 2013 Jul 18;9(7):e1003647. doi: 10.1371/journal.pgen.1003647 (PMC3715433; doi:10.1371/journal.pgen.1003647)
Supplement: Figure S1 — Sin3/Elp3 does not affect histone modification at CESR genes. (A) Stress-dependent H3 acetylation at CESR genes does not require Sin3/Elp3. Cultures of strains 972 (WT) and IV16 (Δsin3/elp3) were treated (+) or not (−) with 1 mM H2O2 for 5 min. ChIP assays were performed using antibodies specific for acetylated Lys9 and Lys14 of histone H3 (H3Ac) or against unmodified C-terminal domain of H3 (H3). The percentage of immuno precipitation of acetylated H3 versus total H3 is indicated (% IP H3Ac/H3). ChIP experiments were performed using primers covering promoter (prom), coding (ORF) and termination (term) sequences of the ctt1 gene. (B) Stress-dependent nucleosome eviction at CESR genes does not require Sin3/Elp3. The same experiment as in A is represented here as the percentage of immuno precipitation of total H3 (% IP total H3). Error bars (SEM) for all ChIP experiments were calculated from biological triplicates. (PDF) [file pgen.1003647.s001.pdf]

**Fernández-Vázquez et al., 2013**
Figures S1

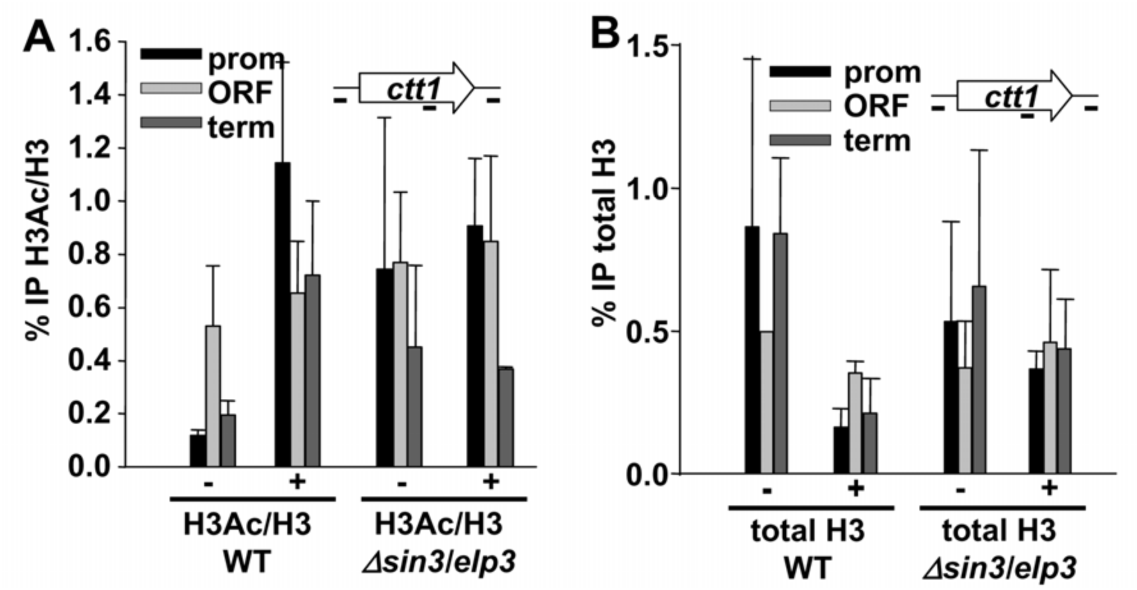


**Figure S1. Sin3/Elp3 does not affect histone modification at CESR genes**. (A) Stress-dependent H3 acetylation at CESR genes does not require Sin3/Elp3. Cultures of strains 972 (WT) and IV16 (Δ*sin3/elp3*) were treated (+) or not (-) with 1 mM $H_2O_2$ for 5 min. ChIP assays were performed using antibodies specific for acetylated Lys9 and Lys14 of histone H3 (H3Ac) or against unmodified C-terminal domain of H3 (H3). The percentage of immuno precipitation of acetylated H3 versus total H3 is indicated (% IP H3Ac/H3). ChIP experiments were performed using primers covering promoter (prom), coding (ORF) and termination (term) sequences of the *ctt1* gene. (B) Stress-dependent nucleosome eviction at CESR genes does not require Sin3/Elp3. The same experiment as in A is represented here as the percentage of immuno precipitation of total H3 (% IP total H3). Error bars (SEM) for all ChIP experiments were calculated from biological triplicates.
