# Supplementary material for: Modification of tRNALys UUU by Elongator Is Essential for Efficient Translation of Stress mRNAs
Source: PLoS Genet. 2013 Jul 18;9(7):e1003647. doi: 10.1371/journal.pgen.1003647 (PMC3715433; doi:10.1371/journal.pgen.1003647)
Supplement: Figure S2 — The bulk tRNA samples of Elongator and Ctu1-Ctu2 mutants do not contain a contaminant inhibitor of tRNA thiolation. Bulk tRNA isolated from strains WT (972), IV16 (Δsin3/elp3), YDH 644 (Δctu1), IV86 (Δctu2), and YDH 254 (Δctu1 Δctu2), were mixed or not as indicated at the top of the panels and analyzed by Northern blot using specific probes against tRNALys UUU, tRNAGln UUG, and tRNAGlu UUC by the APM-gel retardation method. The position of the unmodified (tRNA) or modified (mcm5s2 tRNA) tRNAs is indicated with arrows. (PDF) [file pgen.1003647.s002.pdf]

Fernández-Vázquez et al., 2013
Figures S2

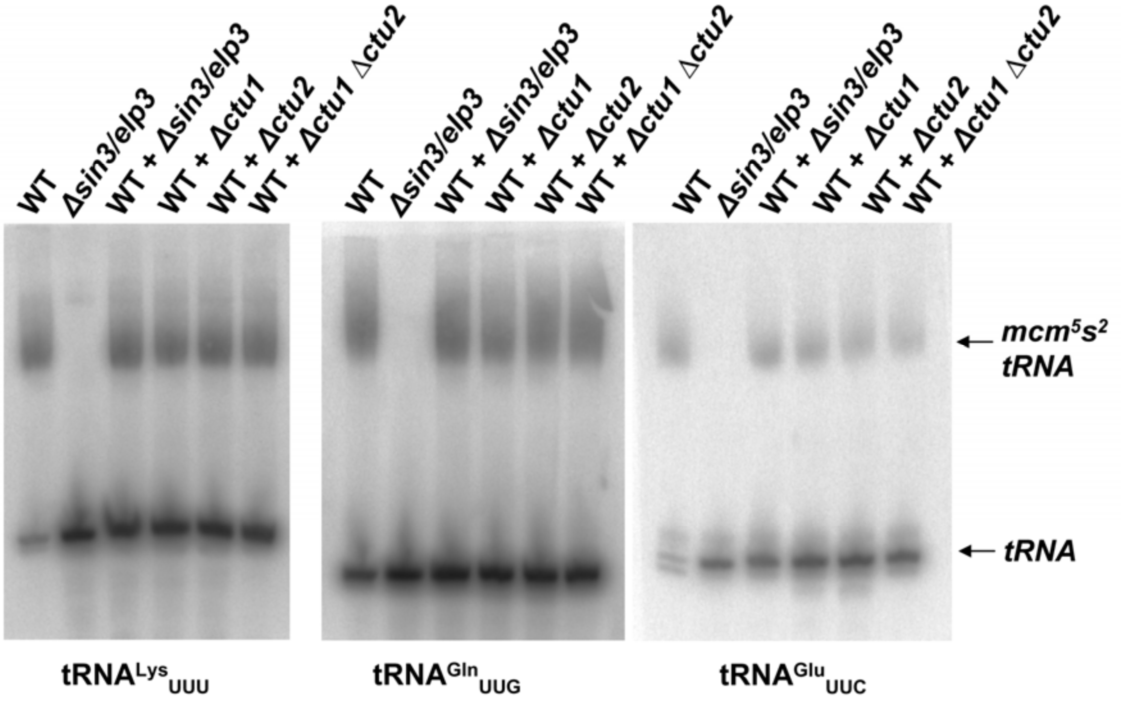

Figure S2. **The bulk tRNA samples of Elongator and Ctu1-Ctu2 mutants do not contain a contaminant inhibitor of tRNA thiolation.** Bulk tRNA isolated from strains WT (972), IV16 (*Δsin3/elp3*), YDH 644 (*Δctu1)*, IV86 (*Δctu2*), and YDH 254 (*Δctu1 Δctu2*), were mixed or not as indicated at the top of the panels and analyzed by Northern blot using specific probes against $tRNA^{Lys}_{UUU}$, $tRNA^{Gln}_{UUG}$, and $tRNA^{Glu}_{UUC}$ by the APM-gel retardation method. The position of the unmodified (*tRNA*) or modified (*mcm^5s^2 tRNA*) tRNAs is indicated with arrows.
