# Supplementary material for: Modification of tRNALys UUU by Elongator Is Essential for Efficient Translation of Stress mRNAs
Source: PLoS Genet. 2013 Jul 18;9(7):e1003647. doi: 10.1371/journal.pgen.1003647 (PMC3715433; doi:10.1371/journal.pgen.1003647)
Supplement: Figure S3 — Over-expression of tRNALys UUU partially supresses the growth defects of Δctu2 upon oxidative stress. Strain JF78 (Δctu2) was transformed with episomal plasmids p465 (tRNALys UUU), p466 (ptRNALys CUU), p467 (ptRNAGln UUG), p468 (ptRNAGlu UUC), or the empty vector pREP.42x. Serial dilutions from cultures of strains 972 (WT), IV86 (Δctu2), and JF78 (Δctu2) transformed with the indicated plasmids were spotted onto rich media plates without (Untreated) or with 1 mM H2O2. (PDF) [file pgen.1003647.s003.pdf]

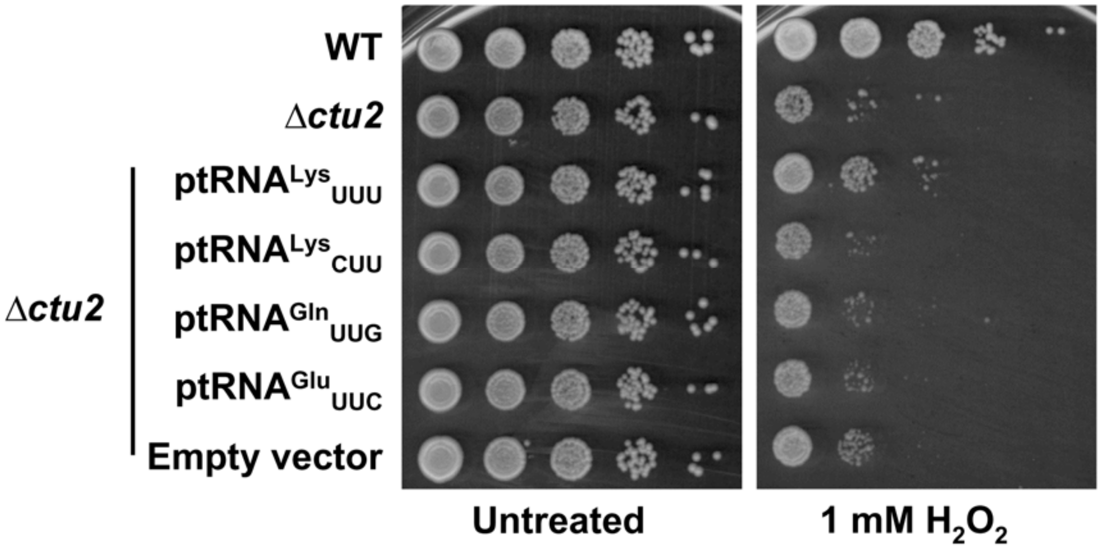

Figure S3. Over-expression of tRNA$^{Lys}_{UUU}$ partially supresses the growth defects of Δ*ctu2* upon oxidative stress. Strain JF78 (*Δctu2*) was transformed with episomal plasmids p465 (tRNA$^{Lys}_{UUU}$), p466 (ptRNA$^{Lys}_{CUU}$), p467 (ptRNA$^{Gln}_{UUG}$), p468 (ptRNA$^{Glu}_{UUC}$), or the empty vector pREP.42x. Serial dilutions from cultures of strains 972 (WT), IV86 (*Δctu2*), and JF78 (*Δctu2*) transformed with the indicated plasmids were spotted onto rich media plates without (Untreated) or with 1 mM H_2O_2.
