# Supplementary material for: Modification of tRNALys UUU by Elongator Is Essential for Efficient Translation of Stress mRNAs
Source: PLoS Genet. 2013 Jul 18;9(7):e1003647. doi: 10.1371/journal.pgen.1003647 (PMC3715433; doi:10.1371/journal.pgen.1003647)
Supplement: Figure S4 — Expression of a synthetic AAA-to-AAG atf1 gene rendered wild-type Atf1 protein levels in Δctu2 mutants. (A and B) Vectors carrying a constitutively expressed wild-type (pHA-atf1′) or a mutated atf1 gene (pHA-atf1 AAG′) were integrated in the chromosomes of wild-type or Δctu2 mutant strains. Rich media cultures of strains JF91 (WT+pHA-atf1′), JF93 (Δctu2+pHA-atf1′), JF94 (WT+pHA-atf1 AAG′) and JF96 (Δctu2+pHA-atf1 AAG′), either untreated (0) or treated with 1 mM H2O2 for the indicated times, were analyzed to determine HA-atf1 mRNA levels by Northern blot using an anti-HA probe (A) or HA-Atf1 protein levels by Western blot using monoclonal antibody against HA (B). The numbers below the Northern or Western blot panels indicate the relative levels of HA-atf1/act1 mRNAs (panel A) or HA-Atf1/tubulin protein levels (panel B), all relative to untreated wild type levels (with an assigned value of 1). (C) Expression of a mutant Atf1 protein does not suppress the growth defects of Δctu2 upon oxidative stress. Empty vector or plasmids carrying a wild-type (pHA-atf1′) or a mutated atf1 gene (pHA-atf1AAG′) were integrated in the chromosomes of wild-type or Δctu2 strains. Cultures from the resulting strains JF88 (WT+empty vector), JF90 (Δctu2+empty vector), JF91 (WT+pHA-atf1′), JF93 (Δctu2+pHA-atf1′), JF94 (WT+pHA-atf1AAG′) and JF96 (Δctu2+pHA-atf1AAG ′) were serially diluted and spotted onto rich media plates without (Untreated) or with 2 mM H2O2. (PDF) [file pgen.1003647.s004.pdf]

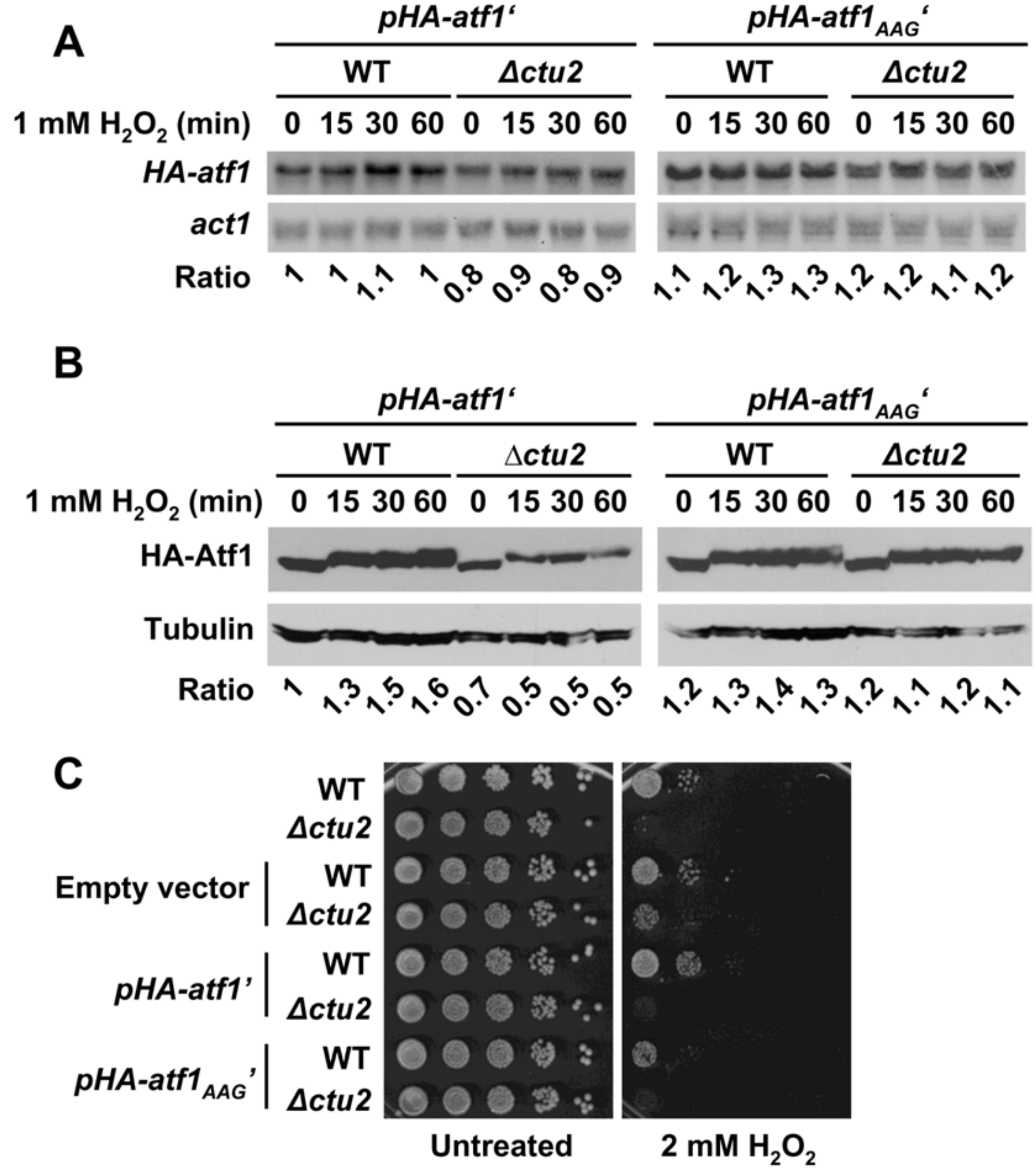

Figure S4. Expression of a synthetic AAA-to-AAG *atf1* gene rendered wild-type Atf1 protein levels in Δ*ctu2* mutants. (A and B) Vectors carrying a constitutively expressed wild-type (p*HA-atf1*') or a mutated *atf1* gene (p*HA-atf1*$_{AAG}$') were integrated in the chromosomes of wild-type or Δ*ctu2* mutant strains. Rich media cultures of strains JF91 (WT + p*HA-atf1*'), JF93 (Δ*ctu2* + p*HA-atf1*'), JF94 (WT + p*HA-atf1*$_{AAG}$') and JF96 (Δ*ctu2* + p*HA-atf1*$_{AAG}$'), either untreated (0) or treated with 1 mM H_2O_2 for the indicated times, were analyzed to determine *HA-atf1* mRNA levels by Northern blot using an anti-HA probe (A) or HA-Atf1 protein levels by Western blot using monoclonal antibody against HA (B). The numbers below the Northern or Western blot panels indicate the relative levels of *HA-atf1/act1* mRNAs (panel A) or HA-Atf1/tubulin protein levels (panel B), all relative to untreated wild type levels (with an assigned value of 1). (C) Expression of a mutant Atf1 protein does not suppress the growth defects of Δ*ctu2* upon oxidative stress. Empty vector or plasmids carrying a wild-type (*pHA-atf1*') or a mutated *atf1* gene (*pHA-atf1*$_{AAG'}$) were integrated in the chromosomes of wild-type or Δ*ctu2* strains. Cultures from the resulting strains JF88 (WT + empty vector), JF90 (Δ*ctu2* + empty vector), JF91 (WT + *pHA-atf1*'), JF93 (*Δctu2* + *pHA-atf1*'), JF94 (WT + *pHA-atf1*$_{AAG'}$) and JF96 (*Δctu2* + *pHA-atf1*$_{AAG'}$) were serially diluted and spotted onto rich media plates without (Untreated) or with 2 mM H_2O_2.
