# Supplementary material for: Modification of tRNALys UUU by Elongator Is Essential for Efficient Translation of Stress mRNAs
Source: PLoS Genet. 2013 Jul 18;9(7):e1003647. doi: 10.1371/journal.pgen.1003647 (PMC3715433; doi:10.1371/journal.pgen.1003647)
Supplement: Figure S5 — Expression of a synthetic AAA-to-AAG atf1 gene replacing the endogenous atf1 locus partially recovered wild-type expression levels of Atf1 protein in cells lacking Elongator or Ctu2. (A and B) The genomic atf1 locus of wild-type, Δsin3/elp3 or Δctu2 strains was replaced with a mutated atf1AAG gene. Rich media cultures of strains JF106 (wild type), JF107 (Δsin3/elp3), JF108 (Δctu2), JF109 (wild type carrying an atf1AAG allele), JF110 (Δsin3/elp3 carrying an atf1AAG allele), and JF111 (Δctu2 carrying an atf1AAG allele), either untreated (0) or treated with 1 mM H2O2 for the indicated times, were analyzed to determine atf1 mRNA levels by Northern blot (A) or Atf1 protein levels by Western blot using polyclonal antibodies against Atf1 (B). The numbers below the Northern or Western blot panels indicate the relative levels of atf1/act1 mRNAs (panel A) or Atf1/tubulin proteins (panel B). (PDF) [file pgen.1003647.s005.pdf]

**Fernández-Vázquez et al., 2013**
Figures S5

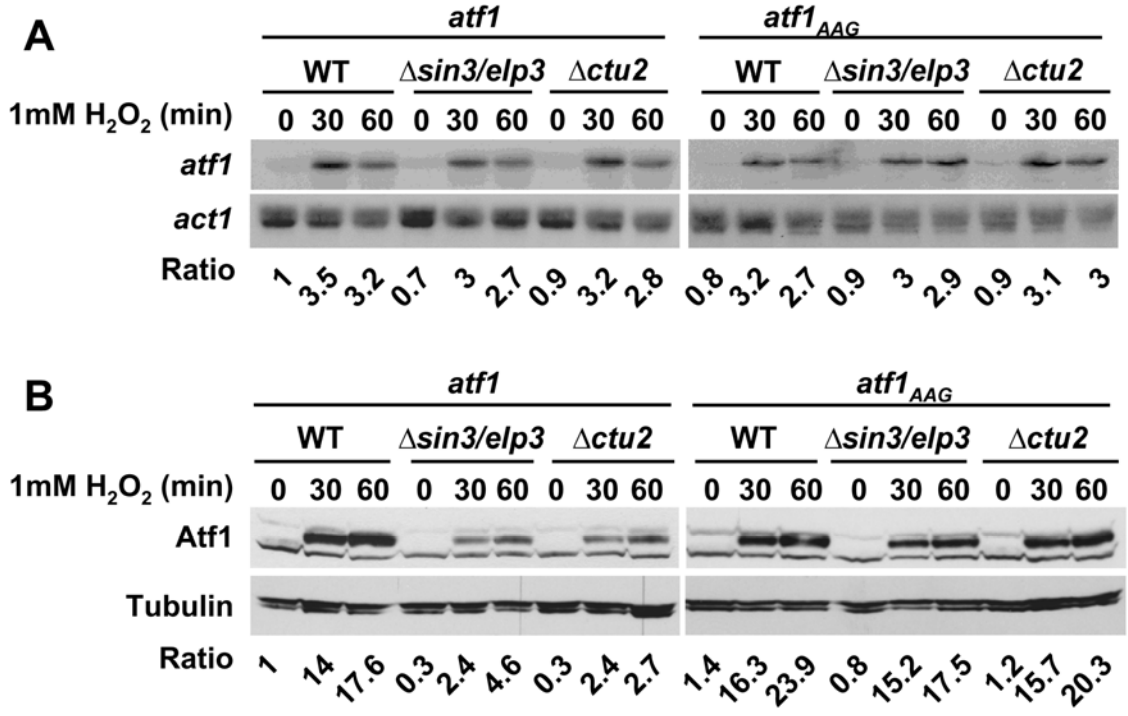


**Figure S5**. **Expression of a synthetic AAA-to-AAG *atf1* gene replacing the endogenous *atf1* locus partially recovered wild-type expression levels of Atf1 protein in cells lacking Elongator or Ctu2**. (A and B) The genomic *atf1* locus of wild-type, Δ*sin3/elp3* or Δ*ctu2* strains was replaced with a mutated $atf1_{AAG}$ gene. Rich media cultures of strains JF106 (wild type), JF107 (Δ*sin3/elp3*), JF108 (Δ*ctu2*), JF109 (wild type carrying an $atf1_{AAG}$ allele), JF110 (Δ*sin3/elp3* carrying an $atf1_{AAG}$ allele), and JF111 (Δ*ctu2* carrying an $atf1_{AAG}$ allele), either untreated (0) or treated with 1 mM $H_2O_2$ for the indicated times, were analyzed to determine *atf1* mRNA levels by Northern blot (A) or Atf1 protein levels by Western blot using polyclonal antibodies against Atf1 (B). The numbers below the Northern or Western blot panels indicate the relative levels of *atf1/act1* mRNAs (panel A) or Atf1/tubulin proteins (panel B).
