# Supplementary material for: Modification of tRNALys UUU by Elongator Is Essential for Efficient Translation of Stress mRNAs
Source: PLoS Genet. 2013 Jul 18;9(7):e1003647. doi: 10.1371/journal.pgen.1003647 (PMC3715433; doi:10.1371/journal.pgen.1003647)
Supplement: Figure S6 — Growth curves of wild-type and tRNA-modifying mutants in minimal medium. (A) Strains 972 (WT), IV16 (Δsin3/elp3), YDH 644 (Δctu1) and IV86 (Δctu2) were grown in minimal medium and the OD600 were recorded at the times indicated. (B) Strains 972 (WT), IV16 (Δsin3/elp3) and JF77 transformed with the indicated plasmids (Δsin3/elp3 tRNA) were grown in minimal medium and the OD600 were recorded at the times indicated. (PDF) [file pgen.1003647.s006.pdf]

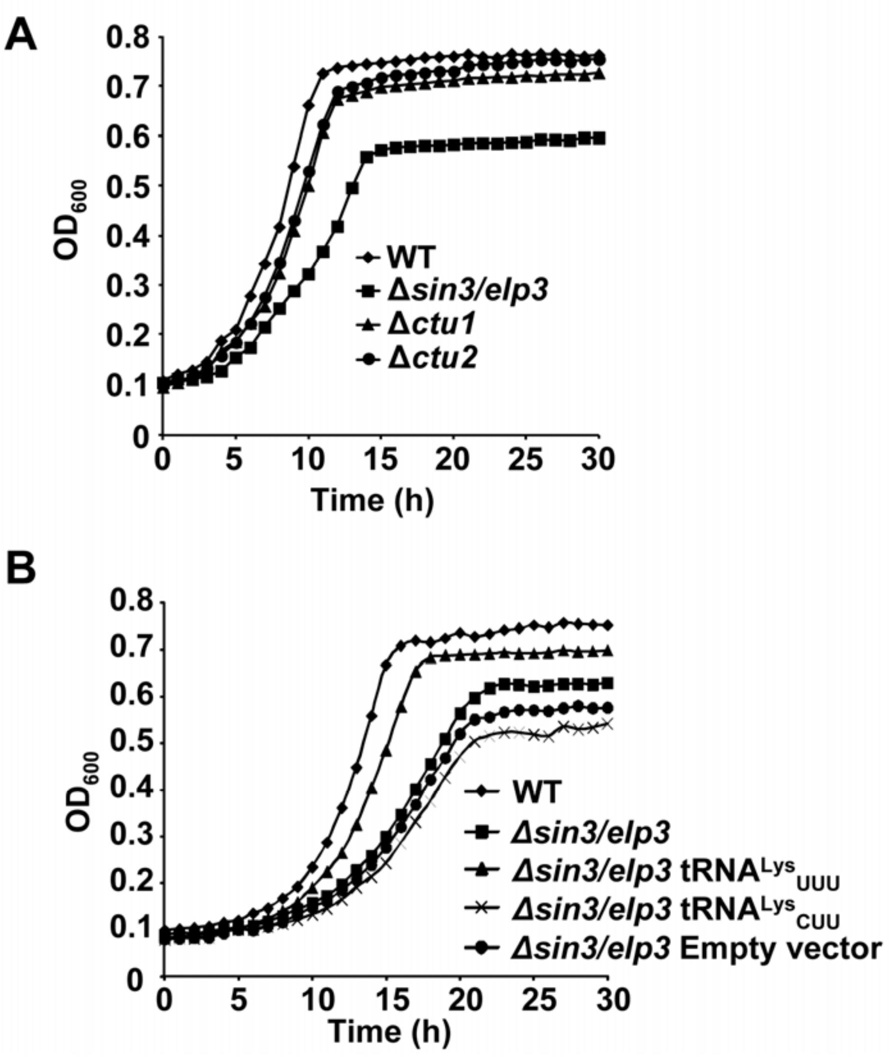

Figure S6. Growth curves of wild-type and tRNA-modifying mutants in minimal medium. (A) Strains 972 (WT), IV16 (Δ*sin3/elp3*), YDH 644 (Δ*ctu1*) and IV86 (Δ*ctu2*) were grown in minimal medium and the OD_{600} were recorded at the times indicated. (B) Strains 972 (WT), IV16 (Δ*sin3/elp3*) and JF77 transformed with the indicated plasmids (Δ*sin3/elp3* tRNA) were grown in minimal medium and the OD_{600} were recorded at the times indicated.
