# Supplementary material for: Modification of tRNALys UUU by Elongator Is Essential for Efficient Translation of Stress mRNAs
Source: PLoS Genet. 2013 Jul 18;9(7):e1003647. doi: 10.1371/journal.pgen.1003647 (PMC3715433; doi:10.1371/journal.pgen.1003647)
Supplement: Table S1 — Proteins that constitute the Elongator complex in S. cerevisiae, and their orthologs in S. pombe. (PDF) [file pgen.1003647.s007.pdf]

Fernández-Vázquez et al., 2013
Tables S1

Table S1. Proteins that constitute the Elongator complex in *S. cerevisiae*, and their orthologs in *S. pombe* (Data from Biobase biological databases).

S. cerevisiae	***S. pombe***
ELP1/IKI3	Iki3
ELP2	SPCC895.06
ELP3	Sin3/Elp3
ELP4	Elp4
ELP5/IKI1	Iki1
ELP6	SPBC3H7.10
KTI12	SPAC30.02C
