# Supplementary material for: Modification of tRNALys UUU by Elongator Is Essential for Efficient Translation of Stress mRNAs
Source: PLoS Genet. 2013 Jul 18;9(7):e1003647. doi: 10.1371/journal.pgen.1003647 (PMC3715433; doi:10.1371/journal.pgen.1003647)
Supplement: Table S2 — Strains used in this study. (PDF) [file pgen.1003647.s008.pdf]

Fernández-Vázquez et al., 2013
Tables S2 (with references)

Table S2. Strains used in this study

Strain	Genotype	Origin
972	*h⁻*	[1]
AV18	*sty1::kanMX6 h⁻*	[2]
MS161	*gcn5::natMX6 h⁺*	[3]
YDH 254	*ctu1::kanMX6 ctu2::kanMX6 ade6 ura4-D18 leu1-32 h⁻*	[4]
YDH 644	*ctu1::kanMX6 h⁻*	[4]
Δctu2	*ctu2::kanMX6 leu1-32 ura4-D18 ade6 h⁺*	[5]
SPK19	*clr3::kanMX6 h⁻*	[6]
MS98	*atf1::natMX6 h⁻*	This work
IV16	*sin3::natMX6 h⁺*	This work
IV66	*iki3::kanMX6 h⁻*	This work
IV68	*SPAC30.02c::kanMX6 h⁻*	This work
IV72	*elp4::kanMX6 h⁻*	This work
IV85	*sin3::kanMX6 leu1-32 h⁻*	This work
IV86	*ctu2::kanMX6 h⁻*	This work
IV87	*ctu2::kanMX6 leu1-32 h⁻*	This work
IV95	*sin3::natMX6 clr3::kanMX6 h⁻*	This work
JF73	*sin3::natMX6 ctu2::kanMX6 h⁻*	This work
JF77	*sin3::kanMX6 leu1-32 ura4-D18 h⁻*	This work
JF78	*ctu2::kanMX6 leu1-32 ura4-D18 h⁻*	This work
HM123	*leu1-32 h⁻*	[7]
JF85	*atf1::ura4 leu1-32 ura4-D18 h⁻*	This work
JF86	*sin3::kanMX6 atf1::ura4 leu1-32 ura4-D18 h⁻*	This work
JF87	*ctu2::kanMX6 atf1::ura4 leu1-32 ura4-D18 h⁻*	This work
JF88	*leu1-32 sty1::HA::leu1 h⁻*	This work
JF89	*sin3::kanMX6 leu1-32 sty1::HA::leu1 h⁻*	This work
JF90	*ctu2::kanMX6 leu1-32 sty1::HA::leu1 h⁻*	This work
JF91	*leu1-32 sty1::HA-atf1::leu1 h⁻*	This work
JF92	*sin3::kanMX6 leu1-32 sty1::HA-atf1::leu1 h⁻*	This work
JF93	*ctu2::kanMX6 leu1-32 sty1::HA-atf1::leu1 h⁻*	This work
JF94	*leu1-32 sty1::HA-*$atf1_{AAG}$*::leu1 h⁻*	This work
JF95	*sin3::kanMX6 leu1-32 sty1::HA-*$atf1_{AAG}$*::leu1 h⁻*	This work
JF96	*ctu2::kanMX6 leu1-32 sty1::HA-*$atf1_{AAG}$*::leu1 h⁻*	This work
JF106	*leu1-32 ura4-D18 h⁻*	This work
JF107	*sin3::kanMX6 leu1-32 ura4-D18 h⁻*	This work
JF108	*ctu2::kanMX6 leu1-32 ura4-D18 h⁻*	This work
JF109	$atf1_{AAG}$ *leu1-32 ura4-D18 h⁻*	This work
JF110	*sin3::kanMX6* $atf1_{AAG}$ *leu1-32 ura4-D18 h⁻*	This work
JF111	*ctu2::kanMX6* $atf1_{AAG}$ *leu1-32 ura4-D18 h⁻*	This work

REFERENCES

1. Leupold U (1970) Genetical methods for *Schizosaccharomyces pombe*. Methods Cell Physiol 4: 169-177.

2. Zuin A, Vivancos AP, Sanso M, Takatsume Y, Ayte J, et al. (2005) The glycolytic metabolite methylglyoxal activates Pap1 and Sty1 stress responses in Schizosaccharomyces pombe. J Biol Chem 280: 36708-36713.

3. Sanso M, Vargas-Perez I, Quintales L, Antequera F, Ayte J, et al. (2011) Gcn5 facilitates Pol II progression, rather than recruitment to nucleosome-depleted stress promoters, in Schizosaccharomyces pombe. Nucleic Acids Res 39: 6369-6379.

4. Dewez M, Bauer F, Dieu M, Raes M, Vandenhaute J, et al. (2008) The conserved Wobble uridine tRNA thiolase Ctu1-Ctu2 is required to maintain genome integrity. Proc Natl Acad Sci U S A 105: 5459-5464.

5. Kim DU, Hayles J, Kim D, Wood V, Park HO, et al. (2010) Analysis of a genome-wide set of gene deletions in the fission yeast Schizosaccharomyces pombe. Nat Biotechnol 28: 617-623.

6. Hansen KR, Burns G, Mata J, Volpe TA, Martienssen RA, et al. (2005) Global effects on gene expression in fission yeast by silencing and RNA interference machineries. Mol Cell Biol 25: 590-601.

7. Moreno S, Klar A, Nurse P (1991) Molecular genetic analysis of fission yeast Schizosaccharomyces pombe. Methods Enzymol 194: 795-823.
